# Telomere length and aging‐related outcomes in humans: A Mendelian randomization study in 261,000 older participants

**DOI:** 10.1111/acel.13017

**Published:** 2019-08-24

**Authors:** Chia‐Ling Kuo, Luke C. Pilling, George A. Kuchel, Luigi Ferrucci, David Melzer

**Affiliations:** ^1^ Department of Community Medicine and Health Care, Connecticut Convergence Institute for Translation in Regenerative Engineering, Institute for Systems Genomics University of Connecticut Health Farmington CT USA; ^2^ Epidemiology and Public Health Group, University of Exeter Medical School, RILD Level 3 Royal Devon & Exeter Hospital Exeter UK; ^3^ Center on Aging, School of Medicine University of Connecticut Farmington CT USA; ^4^ National Institute on Aging Baltimore MD USA

**Keywords:** anti‐aging, cellular senescence, centenarians, frailty, longevity, sarcopenia, TERT, UK Biobank

## Abstract

Inherited genetic variation influencing leukocyte telomere length provides a natural experiment for testing associations with health outcomes, more robust to confounding and reverse causation than observational studies. We tested associations between genetically determined telomere length and aging‐related health outcomes in a large European ancestry older cohort. Data were from *n* = 379,758 UK Biobank participants aged 40–70, followed up for mean of 7.5 years (*n* = 261,837 participants aged 60 and older by end of follow‐up). Thirteen variants strongly associated with longer telomere length in peripheral white blood cells were analyzed using Mendelian randomization methods with Egger plots to assess pleiotropy. Variants in *TERC*, *TERT*, *NAF1*, *OBFC1*, and *RTEL1* were included, and estimates were per 250 base pairs increase in telomere length, approximately equivalent to the average change over a decade in the general white population. We highlighted associations with false discovery rate‐adjusted *p*‐values smaller than .05. Genetically determined longer telomere length was associated with lowered risk of coronary heart disease (CHD; OR = 0.95, 95% CI: 0.92–0.98) but raised risk of cancer (OR = 1.11, 95% CI: 1.06–1.16). Little evidence for associations were found with parental lifespan, centenarian status of parents, cognitive function, grip strength, sarcopenia, or falls. The results for those aged 60 and older were similar in younger or all participants. Genetically determined telomere length was associated with increased risk of cancer and reduced risk of CHD but little change in other age‐related health outcomes. Telomere lengthening may offer little gain in later‐life health status and face increasing cancer risks.

## 1 INTRODUCTION

Telomeres are end fragments of chromosomes consisting of thousands of repeats of the noncoding sequence TTAGGG. Telomeres function to protect chromosome ends against genomic instability. Telomeres shorten with each cell cycle and contribute to replicative senescence when reaching the Hayflick limit (Hayflick & Moorhead, 1961). Telomerase is a ribonucleoprotein complex, which replenishes telomere loss during replication. Telomerase is active at early developmental stages but almost completely inactive in somatic tissues of adults (Collins and Mitchell, 2002). Telomerase activation may treat aging‐related diseases and prolong human lifespan (de Jesus & Blasco, 2013). Previous studies on adult or old mice have shown successes from improving physical function and lifespan without increasing incidence of cancer, but the translation from mice to humans is unknown (de Jesus & Blasco, 2013).

Telomere length is often approximated using leukocyte telomere length, which is easy to extract from blood and highly correlated with telomere length in other tissues (Daniali et al., (2013)). Measured telomere length has been associated with mortality and aging‐related outcomes in humans (Mather, Jorm, Parslow, & Christensen 2011; Sanders & Newman, 2013; Brown, Zhang, Mitchel, & Ailshire, 2018), including cancer (Zhang et al., 2017), cardiovascular disease (Haycock et al., 2014), cognitive function, physical performance such as grip strength, sarcopenia, and frailty (Lorenzi et al., 2018; Zhou et al., 2018), plus biomarkers of lung function, blood pressure, bone mineral density, cholesterol, interleukin 6, and C‐reactive protein. Observational associations cannot be consistently replicated likely due to study populations, measurement methods, and statistical modelling (Sanders & Newman, 2013). In addition, a number of factors may confound observational associations such as sex and race/ethnicity, paternal age at birth, smoking, psychological stress, and other psychosocial, environmental, and behavioral factors (Blackburn, Epel, & Lin, 2015; Starkweather et al., 2014).

Telomere length has a strong inherited genetic component in humans (heritability estimates ranging from 34% to 82% (Broer, Codd, & Nyholt 2013). Mendelian randomization (MR) is a powerful statistical method to evaluate the causal relationship between an exposure and an outcome, under certain assumptions (Davey Smith & Hemani, 2014). Analogous to randomized clinical trials, MR creates groups determined by genotypes, which are inherited at random and are independent of confounding factors. In theory, if the groups are associated with the outcome, the association is independent of confounders and is via the exposure, assuming no pleiotropy is present. MR studies are more robust than observational studies to confounding effects, measurement errors or bias, and reverse causation (i.e., free of downstream effects appearing to be causes).

By applying MR, we were able to study the effect of telomere length on aging, with robustness to confounding effects. To date, 16 inherited genetic variants from genome‐wide association studies (GWAS) have been shown to be strongly associated with human leukocyte telomere length using European‐descent population samples (Haycock et al., 2017). Many of these loci harbor telomerase and telomere‐protective protein genes, including *TERC, TERT, NAF1, OBFC1*, and *RTEL1 *(Codd et al., 2013; Haycock et al., 2017). These variants have been used to perform MR, but the focus was on diseases (Haycock et al., 2017; Zhan et al., 2015). Additionally, previous studies tend to be underpowered due to an insufficiently large sample size for a small percent of variance (2%–3%) explained by the genetic variants (Haycock et al., 2017). The small percent of variance affects the power but not validity of the causal inference, if the genetic variants meet the Mendelian randomization assumptions: (a) associated with telomere length, (b) independent of all confounders for the association between telomere length and the outcome, and (c) independent of the outcome conditional on telomere length and all the confounders (Haycock et al., 2017).

In this study, we investigated causal relationships between telomere length and aging‐related outcomes with the focus on common measures of human aging such as grip strength, frailty, and cognitive function. We analyzed European‐descent participants from UK Biobank, with a wealth of genetic and phenotypic data. This study was not designed to analyze every aging trait in UK Biobank. Instead, we selected traits to cover different aspects of aging, using inputs from senior investigators in the team. Cancer, coronary heart disease, hypertension, and pneumonia were selected as they were common in older adults, but we did not attempt to include every individual disease. Disease‐specific MR associations were reported elsewhere (Haycock et al., 2017). Our project is focused on aging traits and is not powered for diseases that require a longer time to accumulate sufficient cases.

## 2 METHODS

### 2.1 UK Biobank

UK Biobank is a prospective, population‐based study recruiting over 500,000 participants aged 40–70 years in 2006–2010. The study collected extensive genetic and phenotypic data at baseline (recruitment), and the follow‐up is conducted mainly through linkages to death certificates, cancer registries, and hospital records (Bycroft et al., 2018). The DNA was extracted from blood samples and was genotyped using Affymetrix UK BiLEVE Axiom array for the first ~50,000 participants and Affymetrix UK Biobank Axiom array for the remaining cohort—the two arrays sharing over 95% similarity.

### 2.2 Selection of included samples

We focused on European‐descent participants (*n* = 451,433) who were about 90% of the cohort and were identified using genetic principal components analysis, as described in our 2017 publication (Pilling et al., 2017). Pairwise kinship coefficients were calculated using genome‐wide SNP (single nucleotide polymorphism) data and the king software (Manichaikul et al., 2010). One in third‐degree or closer pairs were removed to avoid inflation of associations due to family correlations. Among 379,758 unrelated, European‐descent participants, 168,310 participants were 60 and older at baseline, which increased to 261,837 at the last update. The number of participants younger than 60 was 211,448 at baseline and 117,301 at the last update. By the end of follow‐up, 11,014 of 379,758 participants died.

### 2.3 Aging‐related outcomes

We considered the following aging‐related outcomes: (a) parental lifespan, (b) age‐related diseases and pains, (c) cognitive function, (d) physiological biomarkers, and (e) physical capability. Disease outcomes were updated to February 2016. Other outcomes were measured at baseline, including parental lifespan, pains, cognitive function, physiological biomarkers, and physical capability. Mechanisms underlying the studied health outcomes may differ between middle‐aged and older adults. Age‐related diseases and conditions are more likely to be observed in older adults than in younger adults. We focused on 60 and older adults at measurements (at baseline or at the last follow‐up) for participant's aging phenotypes but included all participants and younger adults in sensitivity analyses. Parental lifespan outcomes were analyzed using all participants only, excluding premature deaths (detailed later). A summary of the aging‐related outcomes, overall and in 40–60 and 60 and older separately, is provided in Table 1.

**Table 1:** A summary of selected aging‐related outcomes in adults younger than 60, 60 and older, and at all ages

Aging‐related outcomes	40–60		60 and older		All participants		Statistical Power^a^
	*n*	Frequency (%) or Mean ± SD	*n*	Frequency (%) or Mean ± SD	*n*	Frequency (%) or Mean ± SD	
Parental lifespan							
Both parents top 10% survival	NA	NA	NA	NA	72,343	6,063 (8%)	0.33
Centenarian status of parents	NA	NA	NA	NA	160,912	2,421 (2%)	0.14
Parents’ age at death (average)	NA	NA	NA	NA	173,628	75.46 ± 7.79	1.00
Father’s age at death	NA	NA	NA	NA	265,834	72.22 ± 11.05	1.00
Mother’s age at death	NA	NA	NA	NA	205,331	77.37 ± 9.83	1.00
Age‐related diseases and pains							
Cancer	117,301	8,478 (7%)	261,837	44,218 (17%)	379,138	52,696 (14%)	1.00
CHD	117,301	3,198 (3%)	261,837	28,491 (11%)	379,138	31,689 (8%)	0.98
Colorectal cancer	117,301	501 (<1%)	261,837	4,161 (2%)	379,138	4,662 (1%)	0.25
Breast cancer (women only)	64,264	2,198 (3%)	139,940	10,184 (7%)	204,204	12,382 (6%)	0.62
Prostate cancer (men only)	53,037	259 (<1%)	121,897	6,792 (6%)	174,934	7,051 (4%)	0.42
Hypertension	117,301	20,004 (17%)	261,837	100,786 (38%)	379,138	120,790 (32%)	1.00
Pneumonia	117,301	2,461 (2%)	261,837	10,776 (4%)	379,138	13,237 (3%)	0.67
Depressed over the last 2 weeks	158,632	11,398 (7%)	137,666	4,982 (4%)	296,298	16,380 (6%)	0.30
Back pain for 3+ months	210,706	35,528 (17%)	167,553	29,989 (18%)	378,259	65,517 (17%)	0.98
Hip pain for 3+ months	210,960	15,204 (7%)	167,691	17,681 (11%)	378,651	32,885 (9%)	0.86
Knee pain for 3+ months	210,825	30,949 (15%)	167,620	31,671 (19%)	378,445	62,620 (17%)	0.98
Frailty index‐49 items (range: 0–49)	175,329	1.72 ± 0.57	137,904	6.29 ± 3.66	313,233	1.78 ± 0.56	1.00
Frailty index excluding two cancer‐related items (range: 0–47)	175,329	1.71 ± 0.57	137,904	6.16 ± 3.62	313,233	1.77 ± 0.57	1.00
Cognitive function							
Reaction time (ms)	210,523	530.97 ± 102.83	166,785	587.77 ± 119.31	377,308	556.08 ± 113.96	1.00
Visual memory errors	211,217	3.75 ± 3.04	168,199	4.56 ± 3.52	379,416	4.11 ± 3.29	1.00
Physiological biomarkers							
FEV1	136,499	3.06 ± 0.75	108,537	2.62 ± 0.7	245,036	2.86 ± 0.76	0.99
FVC	136,499	3.98 ± 0.96	108,537	3.52 ± 0.9	245,036	3.78 ± 0.96	0.99
FEV1/FVC ratio	136,499	0.77 ± 0.06	108,537	0.75 ± 0.07	245,036	0.76 ± 0.06	0.99
Heel BMD (grams/cm^2^)	122,939	0.55 ± 0.13	93,638	0.53 ± 0.14	216,577	0.54 ± 0.14	0.98
Hemoglobin concentration (g/dl)	205,145	14.17 ± 1.26	163,294	14.25 ± 1.18	368,439	14.21 ± 1.23	1.00
Diastolic BP (mm/Hg)	196,987	82.03 ± 10.77	157,729	82.38 ± 10.55	354,716	82.19 ± 10.67	1.00
Systolic BP (mm/Hg)	196,984	135.11 ± 18.2	157,722	145.91 ± 19.77	354,706	139.91 ± 19.66	1.00
Physical capability							
Any fall in the last year	211,020	37,817 (18%)	168,017	36,849 (22%)	379,037	74,666 (20%)	0.99
Sarcopenia	207,785	6,775 (3%)	164,219	14,205 (9%)	372,004	20,980 (6%)	0.78
Low hand grip strength	210,651	16,478 (8%)	167,526	27,178 (16%)	378,177	43,656 (12%)	0.96
Low muscle mass	208,273	70,593 (34%)	164,720	79,773 (48%)	372,993	150,366 (40%)	1.00
Fried frailty index (=frail)	NA	NA	144,645	5,225 (4%)	NA	NA	0.32

Abbreviations: BMD, bone mineral density; BP, blood pressure; CHD, coronary heart disease; n, sample size; SD, standard deviation.

a power to detect an odds ratio of 1.07 or a 0.038 SD change in the outcome per 250 base pairs increase in telomere length (10 additional years of aging approximately) using 60 and older adults for participant’s phenotypes and all participants for parental lifespan outcomes, at the 1% significance level.

#### 2.3.1 Parental lifespan

Parent's lifespan has been used as a surrogate for offspring's lifespan (Pilling et al., 2017). Previous studies have showed that offspring of long‐lived parents are more likely to live longer and have better health outcomes than offspring of short‐lived parents (Dutta et al., 2013). Parental lifespans were collected by touchscreen survey questions, completed by participants at baseline and updated with the follow‐up data. Mother's age at death below 57 and father's age below 46 were considered premature deaths and set to missing in the derivation of parental lifespan outcomes. We used a previously published method (Dutta et al., 2013) to fit normal distributions to father's age at death and mother's age at death, and the early death cutoffs were determined by modal age at death minus 1 standard deviation, which was 57 for mothers and 46 for fathers.

We analyzed fathers who died for father's age at death and mothers who died for mother's age at death. We analyzed parents’ age at death using participants where both parents died, calculated as the average of z‐transformed father's age at death and mother's age at death. The z‐transformation was performed by parent's gender. Additionally, we analyzed “both parents top 10% survival” comparing participants with both parents reaching the top 10% of survival (father reached ≥87 years and mother reached ≥90 years) to those with both parents dead before the age of 80. Similarly, we analyzed “centenarian status of parents” comparing participants where the father reached ≥96 years or the mother reached ≥100 years (top 1% in the 1900 U.S. birth year cohort (Sebastiani, Gurinovich, & Bae, 2017)) to participants where the father died at <90 years and the mother died at <95 years. For both parents top 10% survival and centenarian status of parents, we analyzed participants with long‐lived parents as defined regardless of death status and participants where both parents died before the age cutoff(s). Of note, we excluded participants whose parental lifespan outcomes were not yet known, which may introduce selection bias.

#### 2.3.2 Age‐related diseases and pains

The definition of successful aging mostly includes three components: absence of disease, engagement in life, and maintenance of cognitive and physical functioning (Fiocco & Yaffe, 2010). We considered common diseases and conditions in older adults, including any cancer (excluding nonmelanoma skin cancers), coronary heart disease (CHD: myocardial infarction or angina), hypertension, and pneumonia. At the baseline assessment, participants self‐reported prevalent doctor‐diagnosed diseases. These were combined with hospital admission data (April 1997 to February 2016) to identify participants with diagnoses of multiple relevant diseases. The disease status was confirmed regardless of prevalent cases at baseline or incident cases during follow‐up.

We assessed later‐life onset depression, for which there were no corresponding diagnosis codes, using a survey question at baseline that “Over the past two weeks, how often have you felt down, depressed or hopeless?” with the responses of “Not at all,” “Several days,” “More than half the days,” “Nearly every day,” and “Do not know.” Responses other than “Not at all” were grouped to compare against “Not at all.” “Do not know” and no response were excluded from analyses (83,460 participants excluded, about 22% of the sample at baseline).

Back, hip, and knee pains that had lasted more than 3 months were assessed by the survey questions at baseline, for example, “Have you had back pains for more than 3 months?” with the responses of “Yes,” “No,” “Do not know,” and “Prefer not to answer.” “Do not know,” “Prefer not to answer,” and no response were excluded from analyses. The exclusion rate was low, less than 0.4% (<1,500 participants) across questions.

Frailty was defined as a proportion of accumulated deficits reflecting the health state of an individual. We derived the frailty index developed by Williams, Jylhava, Pedersen, and Hagg (2018) as a measure of frailty, which was validated using UK Biobank data. The frailty index (Williams et al., 2018) scores 49 deficits in a wide range, mostly diseases and pains (sensory, cranial, mental well‐being, infirmity, cardiometabolic, respiratory, musculoskeletal, immunological, cancer, pain, and gastrointestinal). The exact deficits and coding can be found in the supplemental material of the original paper (Williams et al., 2018). We transformed the frailty index (number of deficits) by log(x + 1) function to correct skewness of the distribution where 1 was added to avoid infinite values from zero index values. Additionally, two items related to cancer, any cancer diagnosed and multiple cancers diagnosed, were excluded to create a 47‐item frailty index for sensitivity analyses. About 30,000 participants missed one or more deficits and were excluded from analyses.

#### 2.3.3 Cognitive function

Cognitive function can be examined from the domains including memory, language, visuospatial function, attention, and executive function (Fiocco & Yaffe, 2010). In the present study, we focused on reaction time and visual memory errors. The reaction time was assessed by a symbol matching game similar to the card game snap and was calculated as the average time taken to correctly identify a match. Additionally, visual memory errors were measured as the number of errors that a participant made to complete a pairs matching task where 6 pairs of cards were presented for 3 s beforehand. Reaction time was log‐transformed, and visual memory errors were log(x + 1) transformed to correct skewness of the distributions where 1 was added to avoid infinite values from zero visual memory errors.

#### 2.3.4 Physiological biomarkers

In physiological biomarkers, we included FEV1, FVC, FEV1/FVC ratio, heel bone mineral density, hemoglobin concentration, and blood pressure. These biomarkers have been used for disease diagnoses and were previously found associated with morbidity and mortality. FEV1 and FVC were measured by breath spirometry using a Vitalograph Pneumotrac 6800. Heel bone mineral density was estimated based on the Quantitative Ultrasound Index through the calcaneus. From the index, an estimate is made of bone mineral density in grams/cm^2^.

#### 2.3.5 Physical capability

In physical capability, we selected falls in the last year, sarcopenia, and Fried frailty index. Falls in the last year was assessed by the survey question of “In the last year, have you had any falls?”. The responses included “No falls,” “Only one fall,” “More than one fall,” and “Prefer not to answer.” “Only one fall” and “More than one fall” were combined into “≥1 falls,” and “Prefer not to answer” and no response were excluded (721 participants excluded, 0.19% of the sample at baseline).

Sarcopenia was defined as low hand grip strength and low muscle mass using the definition from the European Working Group on Sarcopenia in Older People (EWGSOP; Cruz‐Jentoft et al., 2010). The hand grip strength in UK Biobank was measured by a Jamar J00105 hydraulic hand dynamometer as the maximal score of measurements from both hands. The skeletal muscle mass was measured by the skeletal muscle mass index (Janssen et al., 2000) where weight and bioelectrical impedance were obtained from a Tanita BC418MA body composition analyzer. A maximal hand grip strength of <30 kg for men and < 20 kg for women was considered low hand grip strength. Similarly, the cutoffs for low muscle mass were 8.87 kg/m^2^ and 6.42 kg/m^2^ for men and women, respectively.

Participants were frail according to the Fried frailty index if meeting three or more of the five criteria: self‐reported weight loss (survey question to ask weight change compared to one year ago), self‐reported exhaustion (survey question to ask frequency of feeling tired or having little energy over the past two weeks), self‐reported slow walking pace (survey question to ask usual walking pace: slow walking pace defined as 3 miles per hour), low hand grip strength, and low physical activity. The lowest 20% of the maximal hand grip strength by sex were considered low hand grip strength and similarly for low physical activity where the total physical activity was assessed by the short version of International Physical Activity Questionnaire (IPAQ; Craig, Marshall, & Sjostrom, 2003). Any missing element led to a missing Fried frailty index, and as a result, 23,665 participants were excluded from analyses.

### 2.4 Genetic variants

We used the 16 SNPs utilized by Haycock et al. 2017 to investigate causal relationships between telomere length and specific diseases. They selected SNPs reported on the GWAS catalog with *p*‐values < 5 × 10^−8^ including top hits from the largest GWAS for telomere length using Europeans (Codd et al., 2013). To supplement the list with additional potential instruments, they added SNPs with *p*‐values < 5 × 10^−8^ in a meta‐analysis of six GWASs (9,190 participants of European ancestry from Mangino et al. (Mangino et al., 2012) with telomere length measured by Southern blotting), as well as other GWAS for SNPs with summary statistics available on the GWAS catalog. SNPs were excluded if they had a minor allele frequency less than 0.05 or significant heterogeneous associations between studies.

Among the 16 SNPs, two were not available in the UK Biobank (rs12696304 and rs9419958); however, they were in high linkage disequilibrium (LD) with other SNPs, which were used as proxies. rs1317082 was dropped because it was in perfect LD (*r* ^2^ = 1) with rs10936599. The correlation between SNPs was modeled in association analyses through a correlation matrix of the square root of *r* ^2^ (*r*). *r* was estimated by LDlink (Machiela & Chanock, 2015) using the CEU samples from Phase 3 (version 5) of the 1,000 Genomes Project (CEU: Utah Residents (CEPH) with Northern and Western European Ancestry).

A final list of 13 SNPs is provided in Table S1 (Supplemental Information), including the regression coefficient estimate (Beta) and standard error (SE) for the association between the effect allele (EA) and telomere length measured by mean leukocyte telomere length in base pairs. Beta, standard deviation (SD) change in telomere length per copy of the effect allele, was estimated using the summary data of Mangino et al. (2012). One SD of telomere length corresponds to approximately 650 base pairs (see Supplemental Online Content of Haycock et al. (2017) and Table 1 in Mangino et al. (2012), equivalent to 26 years of additional aging given that the telomere shortening rate in adults is about 25 base pairs per year (Aviv & Shay, 2018).

### 2.5 Statistical analysis

In the framework of MR, the association between telomere length and an outcome was evaluated using the likelihood‐based method by Burgess, Butterworth, and Thompson (2013). Assuming that the SNPs are valid instrumental variables, the association between these SNPs and an outcome implies a causal relationship between the outcome and telomere length. To be valid instrumental variables, these SNPs must be associated with telomere length, independent of the confounders, and associated with the outcome through their effects on telomere length.

The effect of each SNP on mean leukocyte telomere length (SD change in telomere length per copy of the allele associated with longer telomere length) was previously estimated with adjustment for age, sex, body mass index (BMI), and smoking history (Haycock et al., 2017). The effect of each SNP was estimated using a linear regression model for continuous outcomes and a logistic regression for binary outcomes. The frailty index and outcomes to assess cognitive function were log or log + 1 transformed to meet the normality assumption. All the continuous variables were z‐transformed before association analyses. Age at baseline (for outcomes measured at baseline) or age at the last update (for outcomes continuously updated), sex, assessment center, array type, and the first five genetic principal components were included in the model to adjust for. The adjustment was not exactly the same as that for SNP–telomere length associations, which adjusted for BMI and smoking history additionally. Inclusion of covariates not on telomere length–outcome pathways is not necessary when genetic variants are valid instrumental variables but improves precision of the causal estimate for the effect of telomere length on the outcome. The difference in covariate adjustment in genetic variant–telomere length and genetic variant–outcome associations may bias the causal estimate (Davies, Holmes, & Davey, 2018). However, we performed sensitivity analyses adjusting for BMI and smoking status additionally for genetic variant‐outcome associations and found very similar results (results not shown). The SNP‐exposure (here telomere length) and SNP‐outcome (here aging‐related outcomes) association estimates were used as the MR inputs, that is, log of odds ratio or SD change in the outcome per copy of the allele associated with longer telomere length. Additionally, we performed subgroup analyses by sex using men or women only. For interpretability, the results in terms of an odds ratio or SD change in the outcome were rescaled for an increase of 250 base pairs, equivalent to the average change in telomere length over a decade in the general white population.

### 2.6 Sensitivity analysis

For sensitivity analyses on age‐specific effects, we analyzed participants younger than 60 only and a combined group of mid‐age (40–60) and older adults (≥60). As self‐reported disease status may not be reliable, we analyzed incident cases only, diagnosed during follow‐up for diseases with good admission records, that is, cancer, CHD, and pneumonia. We applied the inverse‐variance weighted (IVW) method assuming a random effects model (Burgess et al., 2013) and MR‐Egger method (Bowden, Davey Smith, & Burgess, 2015) to compare to the results from the likelihood‐based method and to assess pleiotropy.

In MR‐Egger plots, per allele association with an aging‐related outcome (*y*‐axis) was reported as log of odds ratio per effect allele for binary outcomes and SD change per effect allele for continuous outcomes, based on the allele associated with longer telomere length. Similarly, per allele association with mean telomere length was measured by SD change in mean telomere length per effect allele (*x*‐axis). The MR‐Egger method estimated the association between telomere length and an aging‐related outcome by the slope of the linear regression line, reported as log of odds ratio for a binary outcome or SD change in a continuous outcome per effect allele. Additionally, the intercept estimate was compared with zero to test against the null hypothesis of no pleiotropy.

The MR methods were carried out using the *MendelianRandomization *(Yavorska & Burgess, 2017) R package where the LD between SNPs was modeled via a correlation matrix of the square root of *r* ^2^ (*r*). All the statistical analyses were performed in R 3.4.1. We highlight associations with FDR‐adjusted *p*‐values < 5% using adults of 60 and older. Results using 60 and older men or women only, participants younger than 60, and all participants are also provided.

### 2.7 Power analysis

We used the online web tool mRnd (Brion, Shakhbazov, & Visscher, 2013; http://cnsgenomics.com/shiny/mRnd/) to perform MR power analyses. The aging‐related outcomes included binary and continuous outcomes. For binary outcomes, we assumed that the proportion of variance in telomere length explained by the SNPs was 2.23%, which was calculated based on the percent of variance explained by individual, uncorrelated SNPs (Haycock et al., 2017). Power was calculated to detect an odds ratio of 1.2 per SD change in telomere length (~650 base pairs) at the 1% significance level. One percent significance level was used to account for multiple testing. For continuous outcomes, we calculated the power for a 0.1 SD change in the outcome per SD change in telomere length. Power to detect an odds ratio of 1.2 per 650 base pairs is equivalent to power to detect an odds ratio of 1.07 per 250 pairs. Similarly, power to detect a 0.1 SD change per 650 base pairs is equivalent to 0.038 SD change per 250 base pairs. Aging‐related outcomes with <80% power for the effect size were considered low‐powered, including both parents top 10% survival, centenarian status of parents, pneumonia, depression, and Fried frailty (Table 1).

### 2.8 Ethics

UK Biobank received an approval from the UK Biobank Research Ethics Committee (REC; REC reference 11/NW/0382). All the participants provided written informed consent to participate in the study and for their data to be used in future research. This research was conducted using the UK Biobank resource, under the application 14631.

## 3 RESULTS

Among the unrelated Europeans (*n* = 379,758), 168,310 (52% women) participants were 60 and older at baseline (64.12 ± 2.85 years), which increased to 261,837 (53% women, 68.73 ± 4.61 years) by the end of follow‐up (February 2016). A total of 11,014 participants died during follow‐up (the oldest 78 years old), and the mean follow‐up time was 7.5 years (median follow‐up time 7.6 years). A summary of aging‐related outcomes, overall and in 40–60 and 60 and older separately, is provided in Table 1. Mother's lifespan (77.37 ± 9.83 years) was longer than father's lifespan (72.22 ± 11.05 years). A total of 6,063 participants with both parents reaching top 10% of survival were compared to 66,280 participants with both parents dead before the age of 80. Diagnosed disease prevalence tended to be higher in men than in women, but women were more likely to suffer from chronic pains. Physiological functions were similar between men and women except bone mineral density level was much lower in women. Additionally, men had better physical capability than women in general.

### 3.1 Mendelian randomization in participants aged 60 and older

**Figure 1:**
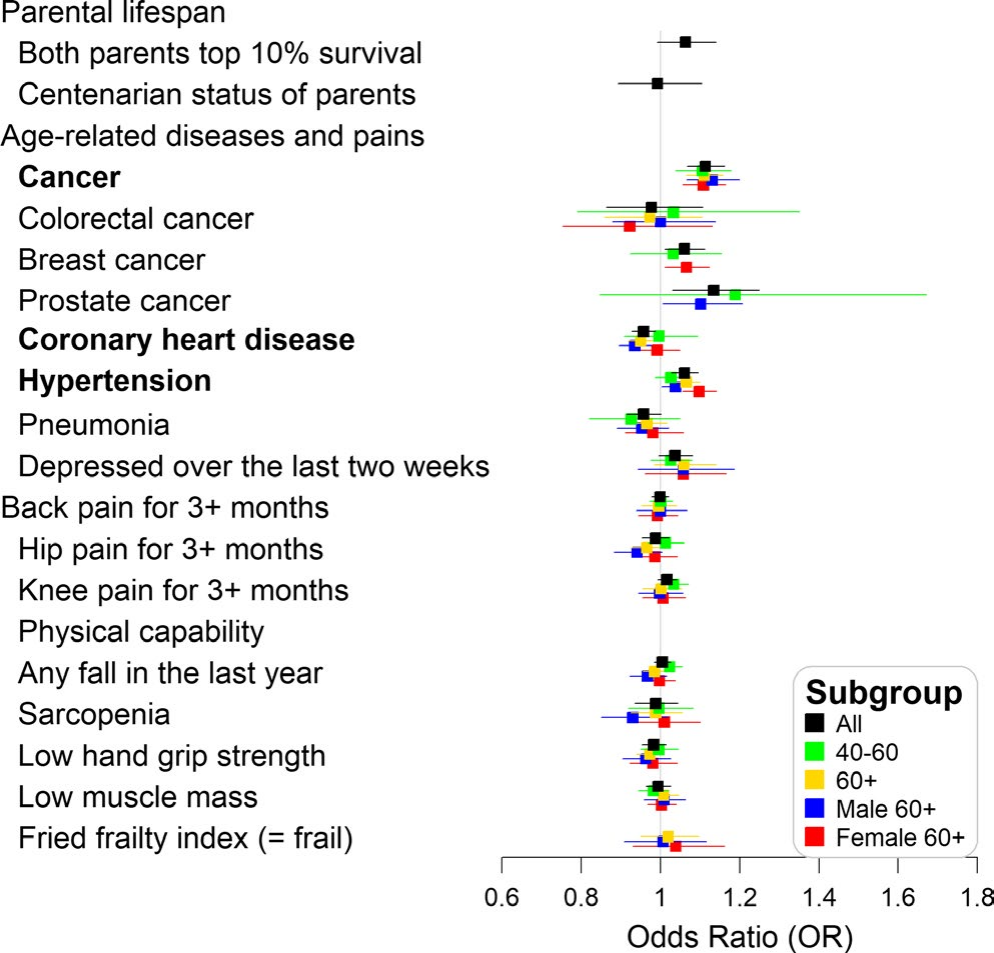
Likelihood‐based Mendelian randomization results for the presence versus absence of an outcome: odds ratio (OR) per 250 base pair increase in telomere length (average change in telomere length over a decade in the general white population). Aging traits highlighted in bold if the false discovery rate‐adjusted *p*‐values < 5% using all participants for parental lifespan outcomes and using participants aged 60 and older for other aging‐related outcomes; All: all participants, 40–60:40 ≤age at measurement <60; 60+: 60 and older at measurement, Male 60+: men 60 and older at measurement, Female 60+: women 60 and older at measurement

**Figure 2:**
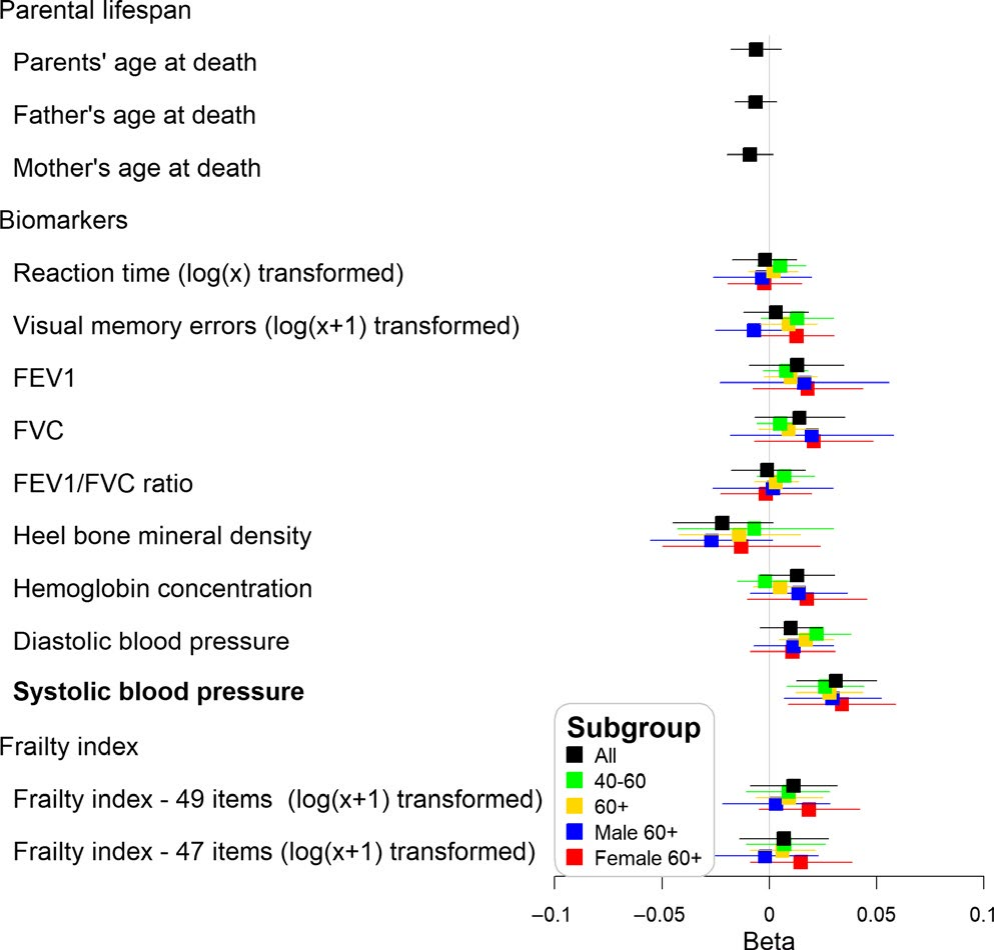
Likelihood‐based Mendelian randomization results for continuous outcomes: SD change (Beta) in the outcome per 250 base pairs (average change in telomere length over a decade in the general white population). Aging traits highlighted in bold if the false discovery rate‐adjusted *p*‐values < 5%, using all participants for parental lifespan outcomes and using participants aged 60 and older for other aging‐related outcomes; All: all participants, 40–60:40 ≤age at measurement <60; 60+: 60 and older at measurement, Male 60+: men 60 and older at measurement, Female 60+: women 60 and older at measurement

Genetically increased telomere length was associated with higher odds of cancer (OR = 1.11, 95% CI: 1.06–1.16) and hypertension (OR = 1.06, 95% CI: 1.03–1.10) per 250 base pair increase in telomere length (Figure 1). Both traits had similar effect sizes in men and women. Genetically increased telomere length was protective for CHD (OR = 0.95, 95% CI: 0.92–0.98), and the effect was largely driven by men (OR = 0.94, 95% CI: 0.89–0.98) with weak evidence for an association found in women (OR = 0.99, 95% CI: 0.94–1.05; Figure 1). Additionally, systolic blood pressure was increased by 0.031 SD (0.61 mm Hg, 95% CI: 0.26–0.99 mm Hg) per 250 base pair increase in telomere length, with very similar effect sizes in men and women (Figure 2).

Associations with other outcomes did not reach the FDR‐adjusted significance level. Suggestive trends included the following. Genetically increased telomere length was associated with the likelihood of being depressed (OR = 1.06, 95% CI: 0.98–1.14) and increased longevity of parents (both parents top 10% survival with OR = 1.06, 95% CI: 0.99–1.14), whereas pneumonia (OR = 0.95, 95% CI: 0.89–1.02), hip pain (OR = 0.94, 95% CI: 0.88–1.00), and sarcopenia (OR = 0.93, 95% CI: 0.85–1.02) in men were negatively associated with telomere length (Figure 1). Genetically determined telomere length was minimally associated with parental lifespan, centenarian status of parents, cognitive function, or physical performance including falls, grip strength, muscle mass, and frailty.

### 3.2 Sensitivity analysis using 40 to 60 years old and all participants

The results using all participants or participants aged 40–60 only were mostly similar to the results of adults aged 60 and older (Figures 1 and 2). However, the associations with CHD and hypertension were stronger in older adults than younger adults (Figure 1). We analyzed incident cases during follow‐up only using 60 and older adults for the disease outcomes of cancer, CHD, and pneumonia. The effect sizes were very similar for CHD and pneumonia (CHD: OR = 0.95, 95% CI: 0.90–1.00; pneumonia: OR = 0.95, 95% CI: 0.88–1.03). A slightly lower cancer risk was found using incident cases only (OR = 1.09, 95% CI: 1.04–1.13) than from participants ever diagnosed with cancer (OR = 1.11, 95% CI: 1.06–1.16), compared to those cancer‐free.

**Figure 3:**
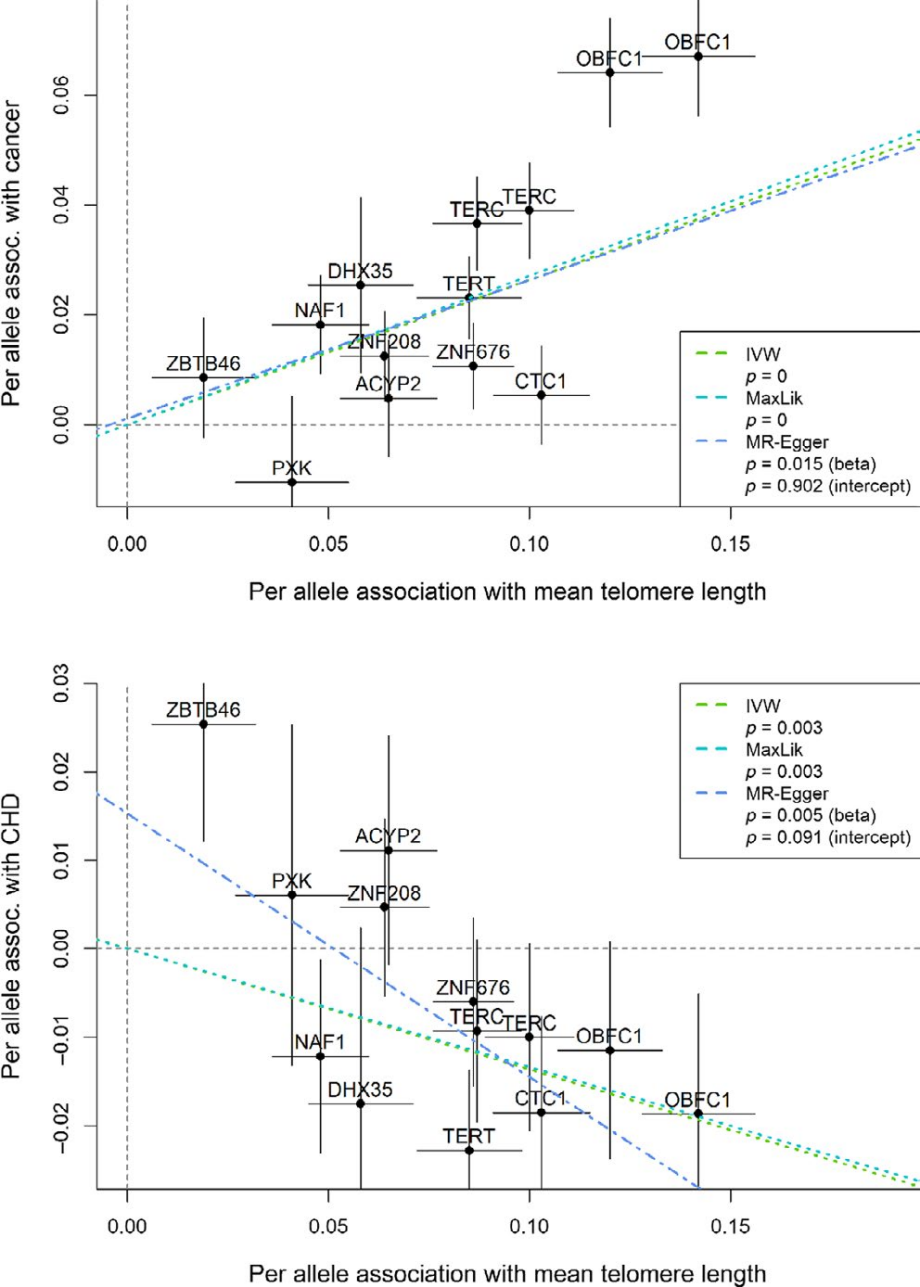
Mendelian randomization sensitivity analysis results for cancer (upper) and CHD (lower). Per allele association with cancer: log of odds ratio for cancer per effect allele, allele associated with longer telomere length; per allele association with CHD: log of odds ratio for coronary heart disease per effect allele; per allele association with mean telomere length: SD change in mean telomere length per effect allele. Inverse‐variance weighted (IVW), likelihood‐based (MaxLik), and MR‐Egger (beta) *p*‐values for associations with telomere length and MR‐Egger (intercept) for pleiotropy

**Figure 4:**
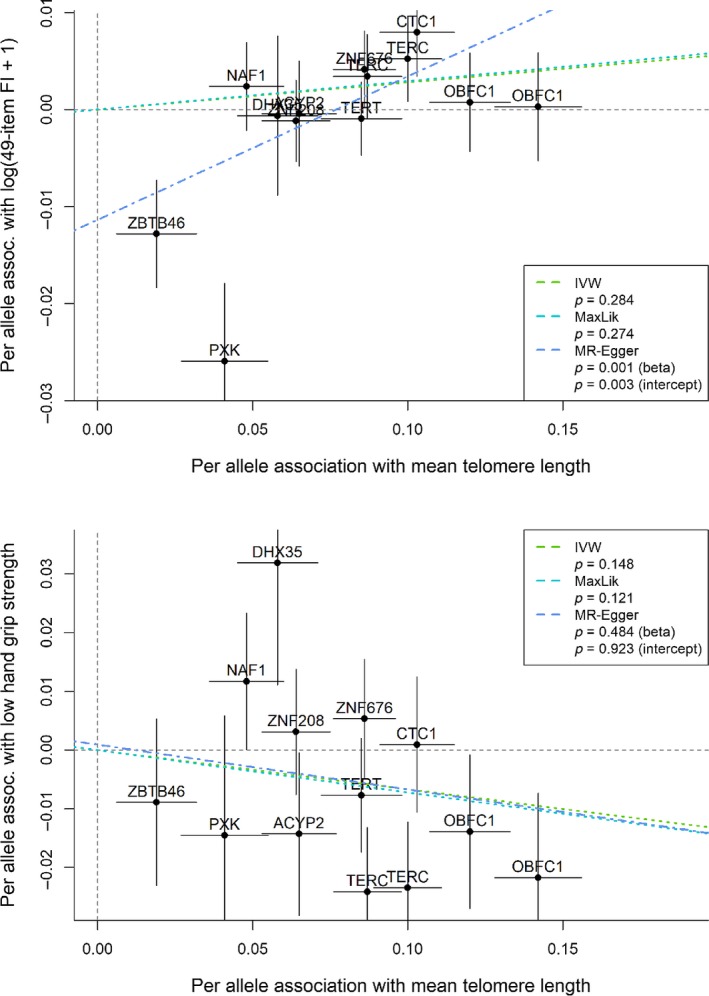
Mendelian randomization sensitivity analysis results for low hand grip strength (upper) and the 49‐item frailty index (lower). Per allele association with low hand grip strength: log of odds ratio for low hand grip strength per effect allele, allele associated with longer telomere length; per allele association with log (49‐item frailty index +1): SD change in log (49‐item frailty index +1) per effect allele; per allele association with mean telomere length: SD change in mean telomere length per effect allele. Inverse‐variance weighted (IVW), likelihood‐based (MaxLik), and MR‐Egger (beta) *p*‐values for associations with telomere length and MR‐Egger (intercept) for pleiotropy

We performed sensitivity analyses using the IVW and MR‐Egger methods. The likelihood‐based method and the IVW method gave very similar results. We used the MR‐Egger method to assess pleiotropy (Bowden et al., 2015). There was little evidence for pleiotropy in the associations with cancer, CHD (Figure 3), or hypertension (MR‐Egger_plots.pdf Data S1 in Supplemental Information). The MR‐Egger plot for systolic blood pressure (MR‐Egger_plots.pdf Data S1 in Supplemental Information) suggested pleiotropy and little association between telomere length variants and systolic blood pressure. Regarding common aging measures, for example, hand grip strength and the 49‐item frailty index (Figure 4), a decreasing trend was found consistently across methods and there was little evidence of pleiotropy for low hand grip strength. The MR‐Egger method suggested an association with the 49‐item frailty index; however, the association came with pleiotropy, and the IVR and likelihood‐based method produced minimal associations.

For more details, MR association results using the likelihood‐based and other methods were provided in Table S2 and “MR‐Egger_plots.pdf” for 60 and older, Table S3 for 60 and older men, Table S4 for 60 and older women, Table S5 for participants younger than 60, and Table S6 for all participants.

## 4 DISCUSSION

We have tested associations between genetic variants linked to telomere length and a range of health outcomes focused on human aging. We studied a large sample of participants aged 60–70, followed up for a mean of 7.5 years, with good power to detect associations. We found that variants associated with longer telomeres were associated with cancer, confirming previous findings. We also found associations with higher blood pressure and reduced risk of CHD, also previously reported. However, associations with common measures of human aging, including parental lifespan, two cognitive measures and two muscle measures, were all not significant at the FDR‐adjusted level.

Our results are similar to the previously reported MR associations between genetically increased telomere length and increased risk of cancer, hypertension, and decreased risk of CHD (Hamad, Walter, & Rehkopf, 2016; Haycock et al., 2017; Helby, Nordestgaard, Benfield, & Bojesen, 2017). Compared to the MR associations reported by Hamad et al. (2016) using the Health Retirement Study (HRS) data, the OR per 100 base pairs (unit used in Hamad et al., 2016) for cancer was 1.04 (95% CI: 1.03–1.06) in UK Biobank and 1.04 (95% CI: 0.97–1.11) in HRS (*n* = 3,734); additionally, OR = 1.03 (95% CI: 1.01–1.04) for hypertension in UK Biobank and OR = 1.04 (95% CI: 0.96–1.12) in HRS, and OR = 0.98 (95% CI: 0.97–0.99) for CHD in UK Biobank and OR = 0.94 (95% CI: 0.88–1.01) for heart disease in HRS. Also, we compared the depression results, self‐evaluated depression in UK Biobank (OR = 1.02, 95% CI: 0.99–1.05), and self‐reported, doctor‐told depression problems in HRS (OR = 1.00, 95% CI: 0.97–1.03).

Our findings echoed previous observational studies although the reported effect sizes may not be comparable due to scaling methods. Previous meta‐analyses showed that longer telomere length was associated with cancer risk (OR = 1.086, 95% CI: 0.952–1.238; Zhang et al., 2017) and protective for coronary heart disease (OR = 1.42, 95% CI: 1.17–1.73) comparing the shortest versus longest third of telomere length (Haycock et al., 2014). Longer telomere length was associated with reduced risk of pneumonia (Helby et al., 2017) and minimal associations were found with chronic pains (Steward, Morgan, Espinosa, Turk, & Patel, 2017), anemia and other hematological parameters (Den Elzen et al., 2011), cognitive function (Brown et al., 2018) and physical measures including lung function (Brown et al., 2018), fracture (Sanders et al., 2009), bone mineral density (Sanders et al., 2009), as well as sarcopenia, and frailty (Lorenzi et al., 2018). The observational associations with systolic and diastolic blood pressures were minimal in HRS (Brown et al., 2018) and National Health and Nutrition Examination Survey (NHANES) study (Rehkopf et al., 2016). Both studies reported an increasing trend in systolic blood pressure with genetically increased telomere length. In our study, the mean change in systolic blood pressure per 250 base pairs was estimated to be 0.61 mm Hg (95% CI: 0.26–0.99 mm Hg), which appears too small to have clinical implications, and the association may not be causal, due to pleiotropy, suggested by the MR‐Egger plot (Figure 3). In short, we found associations with cancer and CHD but not with cognitive and physical function. The associations with cancer may be due to longer telomeres allowing extended cell proliferation or delaying senescence (de Jesus & Blasco, 2013). Other pathways to cellular senescence such as DNA damage may play more important roles than telomere shortening in aging (Anderson, Lagnado, & Maggiorani, 2019).

Genetically increased telomere length was not associated with parents’ survival. Parental lifespan outcomes are surrogates for the participant's survival, as participants in UK Biobank were too young at the end of follow‐up (mean age 64.24 years). Early deaths tend to be driven by diseases rather than normal aging (58% of deaths due to cancer), and more work is required on mortality outcomes with longer follow‐ups.

The genetic variants used in MR were associated with mean leukocyte telomere length in general population samples. These genetic variants may not be ideal if aging is related more to shortest telomere length (Blackburn et al., 2015), although these two measures are likely to be correlated. There is evidence that telomere length at newborn is more predictive than that in adulthood for lifespan (Aviv & Shay, 2018). While the genetic variants were identified using adult samples, the associations were adjusted for demographics and exposures including age, sex, BMI, and smoking history (Haycock et al., 2017).

Our study inevitably has limitations: We have studied Europeans aged 60–70 in UK Biobank, where participants tend to be relatively healthy volunteers, with somewhat lower health risks than the general population (Fry et al., 2017). As a result, prevalence estimated using UK Biobank data may not be generalizable to UK and other cohorts. The causal estimate for the effect of telomere length on an aging‐related outcome could potentially be biased if UK Biobank participation was strongly associated with telomere length and aging‐related outcomes (Munafo, Tilling, Taylor, Evans, & Davey, 2018). We do not have data of those who declined to participate in UK Biobank. Indirectly, we tested for the association between the genetic risk score associated with longer telomere length and participation of the MRI imaging substudy or online diet questionnaires. We found that the genetic risk score was not associated with either participation, which suggested that our results may not be greatly impacted by selection into UK Biobank. People with shorter telomeres may die earlier, introducing survivor bias into the analyses of 60‐plus‐year‐olds, but our analyses in the 40‐ to 60‐year‐olds produced very similar results. This study is not well powered to study longevity of parents and rare diseases and conditions given a short period of follow‐up time. Additionally, we have studied baseline measures of cognitive and physical function, as data on repeat measures are available in only a small percentage of participants. Also, measured telomere length was not available to compare with the genetic variants studied, although the variants have the advantage of being less susceptible to confounding and reverse causation than observational studies.

## 5 CONCLUSIONS

In European ancestry 60‐ to 70‐year‐olds followed for 7.5 years, those inheriting more variants linked to longer telomeres were protected from cardiovascular heart disease but did not have better healthy aging measures, with no better cognitive function, grip strength, sarcopenia, or falls. The presence of a risk of excess cancer in those with genetically longer telomeres poses a major hurdle in harnessing telomere lengthening to prolong human lifespan. Our findings thus do not suggest advantages in lengthening telomeres to improve human aging outcomes.

## CONFLICT OF INTEREST

None declared.
