# Supplementary material for: Telomere length and aging‐related outcomes in humans: A Mendelian randomization study in 261,000 older participants
Source: Aging Cell. 2019 Aug 24;18(6):e13017. doi: 10.1111/acel.13017 (PMC6826144; doi:10.1111/acel.13017)
Supplement: Supplementary file 7 [file ACEL-18-e13017-s007.pdf]

MR-Egger plots

Figure #	Figure Title	Page #
1	Per allele association with any fall in the last year	1
2	Per allele association with back pain for 3+ months	1
3	Per allele association with both parents top 10% survival	2
4	Per allele association with breast cancer	2
5	Per allele association with cancer excluding non-melanoma skin cancers	3
6	Per allele association with centenarian status of parents	3
7	Per allele association with colorectal cancer	4
8	Per allele association with coronary heart disease (CHD)	4
9	Per allele association with depressed over the last two weeks	5
10	Per allele association with diastolic blood pressure	5
11	Per allele association with father's age at death	6
12	Per allele association with FEV1	6
13	Per allele association with FEV1/FVC ratio	7
14	Per allele association with frailty index - 47 items (log(x+1) transformed)	7
15	Per allele association with frailty index - 49 items (log(x+1) transformed)	8
16	Per allele association with Fried frailty index (= frail)	8
17	Per allele association with FVC	9
18	Per allele association with heel bone mineral density	9
19	Per allele association with hemoglobin concentration	10
20	Per allele association with hip pain for 3+ months	10
21	Per allele association with hypertension	11
22	Per allele association with knee pain for 3+ months	11
23	Per allele association with low hand grip strength	12
24	Per allele association with low muscle mass	12
25	Per allele association with mother's age at death	13
26	Per allele association with parents' age at death	13
27	Per allele association with pneumonia	14
28	Per allele association with prostate cancer	14
29	Per allele association with reaction time (log(x) transformed)	15
30	Per allele association with sarcopenia	15
31	Per allele association with systolic blood pressure	16
32	Per allele association with visual memory errors (log(x+1) transformed)	16

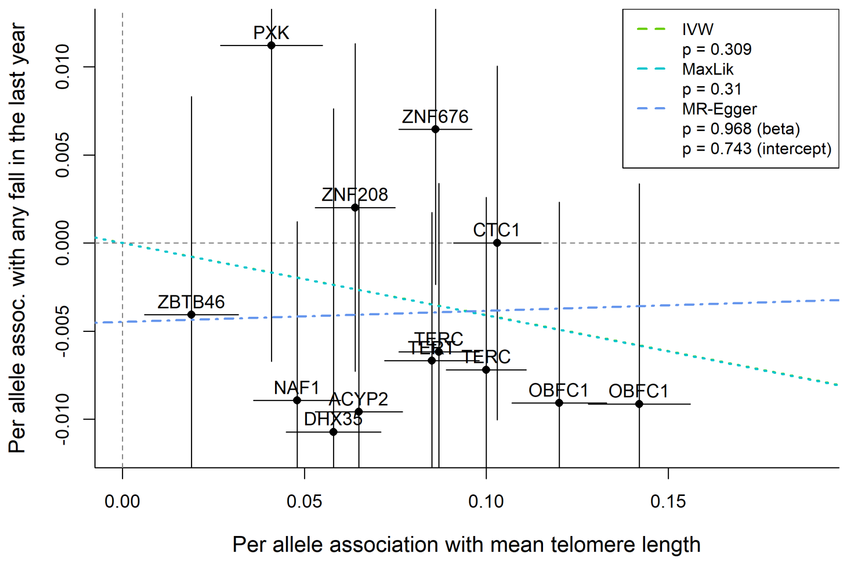

FIG. 1: Per allele association with any fall in the last year: log of odds ratio for any fall in the last year per effect allele, allele associated with longer telomere length; Per allele association with mean telomere length: SD change in mean telomere length per effect allele. Inverse-variance weighted (IVW), likelihood-based (MaxLik), and MR-Egger (beta) p-values for associations with telomere length and MR-Egger (intercept) for pleiotropy.

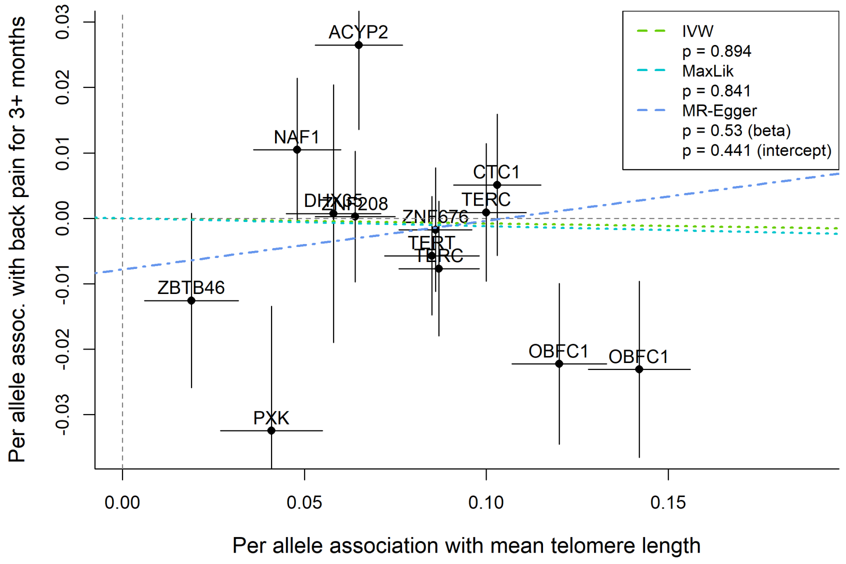

FIG. 2: Per allele association with back pain for 3+ months: log of odds ratio for back pain for 3+ months per effect allele, allele associated with longer telomere length; Per allele association with mean telomere length: SD change in mean telomere length per effect allele. Inverse-variance weighted (IVW), likelihood-based (MaxLik), and MR-Egger (beta) p-values for associations with telomere length and MR-Egger (intercept) for pleiotropy.

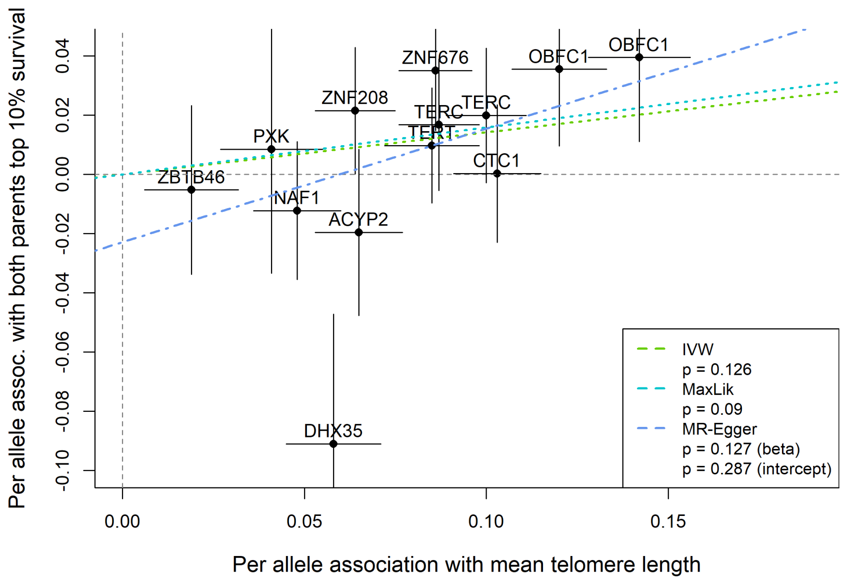

FIG. 3: Per allele association with both parents top 10% survival: log of odds ratio for both parents top 10% survival per effect allele, allele associated with longer telomere length; Per allele association with mean telomere length: SD change in mean telomere length per effect allele. Inverse-variance weighted (IVW), likelihood-based (MaxLik), and MR-Egger (beta) p-values for associations with telomere length and MR-Egger (intercept) for pleiotropy.

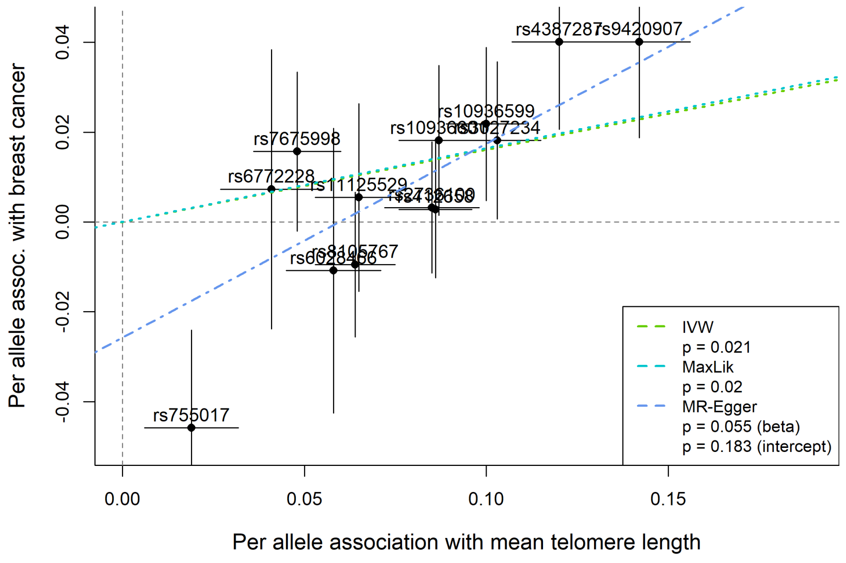

FIG. 4: Per allele association with breast cancer: log of odds ratio for breast cancer per effect allele, allele associated with longer telomere length; Per allele association with mean telomere length: SD change in mean telomere length per effect allele. Inverse-variance weighted (IVW), likelihood-based (MaxLik), and MR-Egger (beta) p-values for associations with telomere length and MR-Egger (intercept) for pleiotropy.

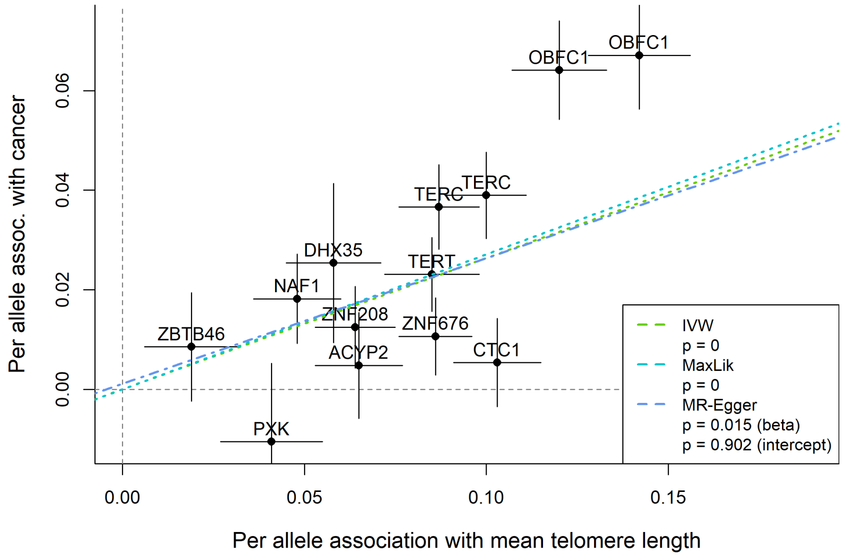

FIG. 5: Per allele association with cancer: log of odds ratio for cancer excluding non-melanoma skin cancers per effect allele, allele associated with longer telomere length; Per allele association with mean telomere length: SD change in mean telomere length per effect allele. Inverse-variance weighted (IVW), likelihood-based (MaxLik), and MR-Egger (beta) p-values for associations with telomere length and MR-Egger (intercept) for pleiotropy.

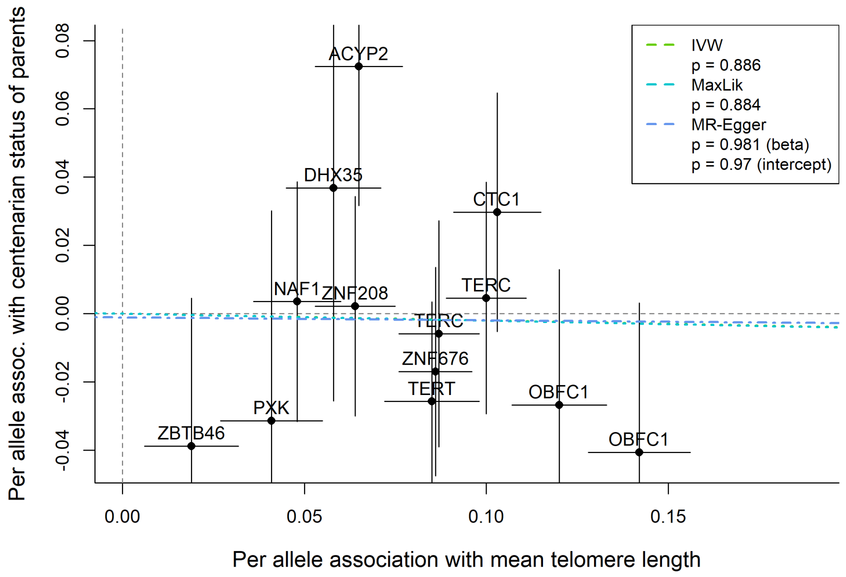

FIG. 6: Per allele association with centenarian status of parents: log of odds ratio for centenarian status of parents per effect allele, allele associated with longer telomere length; Per allele association with mean telomere length: SD change in mean telomere length per effect allele. Inverse-variance weighted (IVW), likelihood-based (MaxLik), and MR-Egger (beta) p-values for associations with telomere length and MR-Egger (intercept) for pleiotropy.

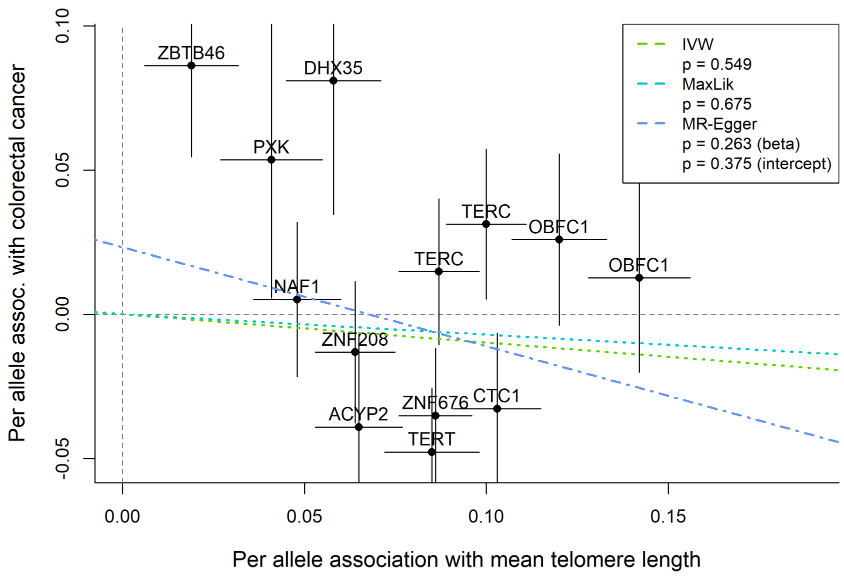

FIG. 7: Per allele association with colorectal cancer: log of odds ratio for colorectal cancer per effect allele, allele associated with longer telomere length; Per allele association with mean telomere length: SD change in mean telomere length per effect allele. Inverse-variance weighted (IVW), likelihood-based (MaxLik), and MR-Egger (beta) p-values for associations with telomere length and MR-Egger (intercept) for pleiotropy.

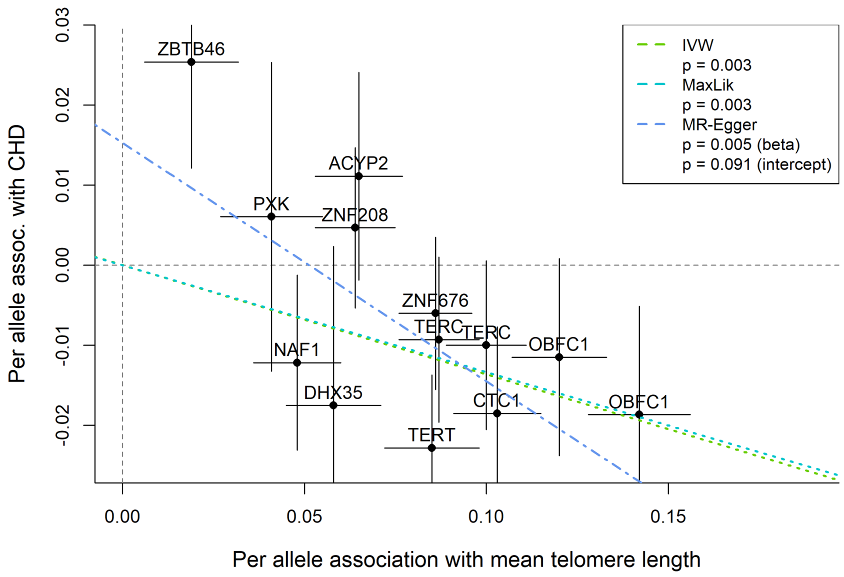

FIG. 8: Per allele association with CHD: log of odds ratio for coronary heart disease per effect allele, allele associated with longer telomere length; Per allele association with mean telomere length: SD change in mean telomere length per effect allele. Inverse-variance weighted (IVW), likelihood-based (MaxLik), and MR-Egger (beta) p-values for associations with telomere length and MR-Egger (intercept) for pleiotropy.

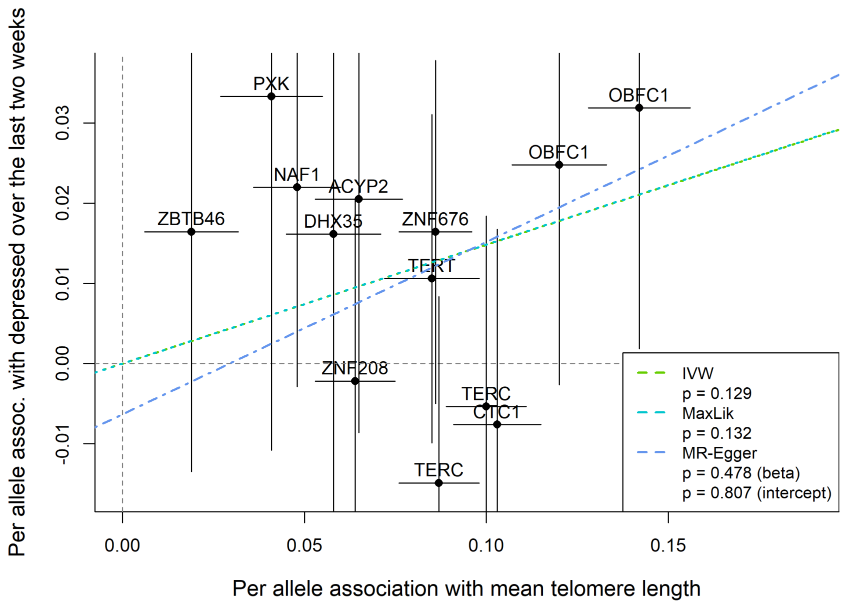

FIG. 9: Per allele association with depression over the last two weeks: log of odds ratio for depression over the last two weeks per effect allele, allele associated with longer telomere length; Per allele association with mean telomere length: SD change in mean telomere length per effect allele. Inverse-variance weighted (IVW), likelihood-based (MaxLik), and MR-Egger (beta) p-values for associations with telomere length and MR-Egger (intercept) for pleiotropy.

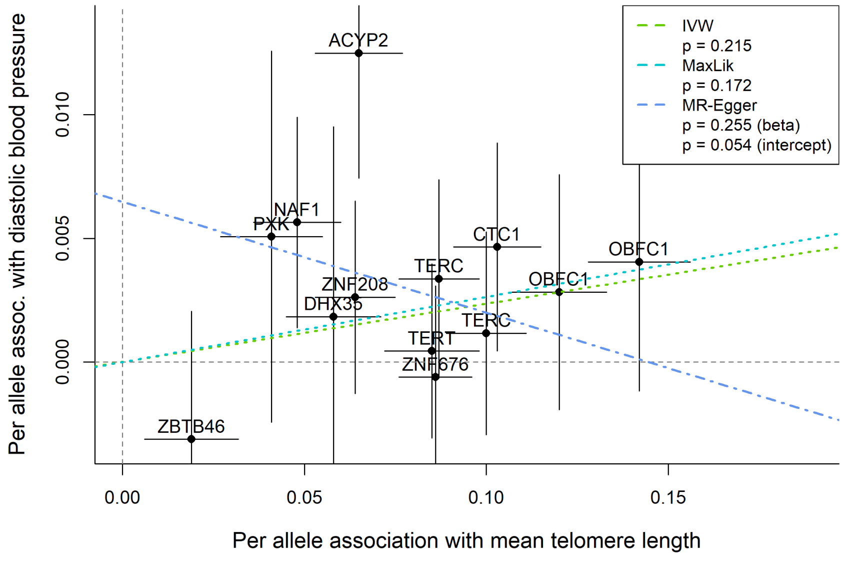

FIG. 10: Per allele association with diastolic blood pressure: log of odds ratio for diastolic blood pressure per effect allele, allele associated with longer telomere length; Per allele association with mean telomere length: SD change in mean telomere length per effect allele. Inverse-variance weighted (IVW), likelihood-based (MaxLik), and MR-Egger (beta) p-values for associations with telomere length and MR-Egger (intercept) for pleiotropy.

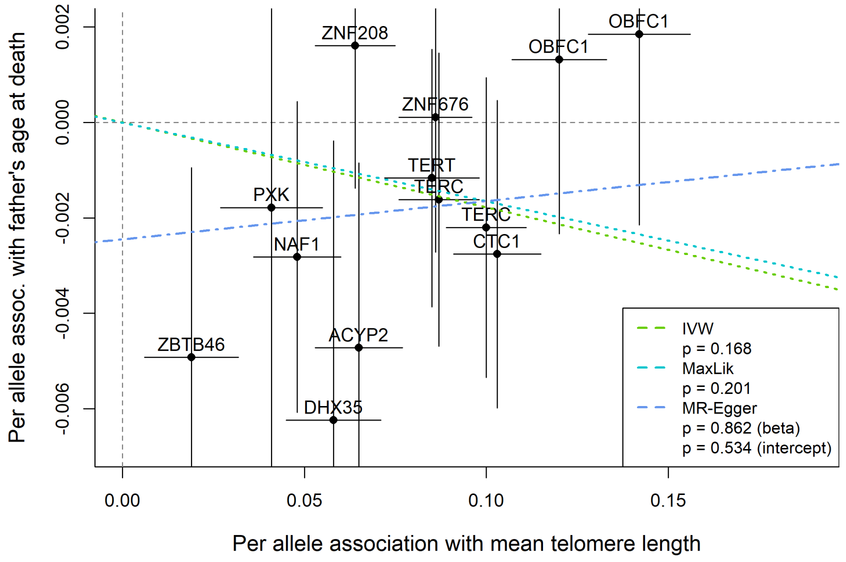

FIG. 11: Per allele association with father's age at death: log of odds ratio for father's age at death per effect allele, allele associated with longer telomere length; Per allele association with mean telomere length: SD change in mean telomere length per effect allele. Inverse-variance weighted (IVW), likelihood-based (MaxLik), and MR-Egger (beta) p-values for associations with telomere length and MR-Egger (intercept) for pleiotropy.

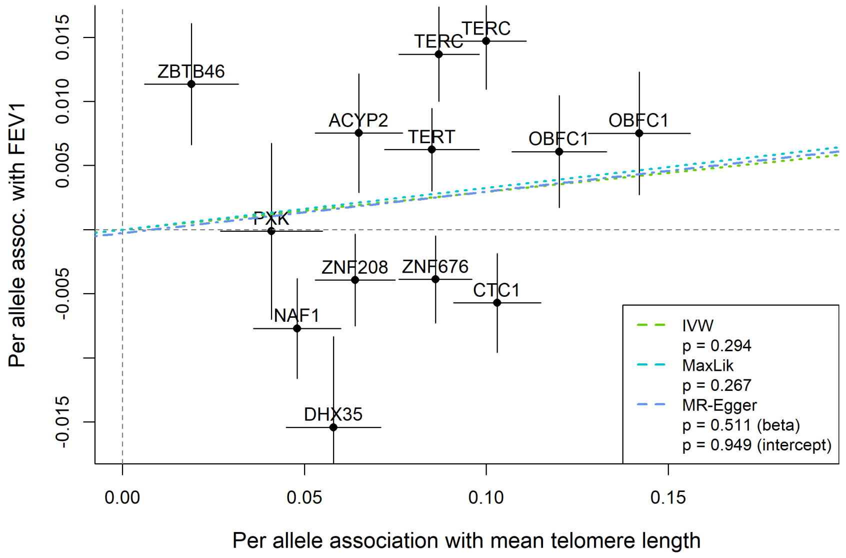

FIG. 12: Per allele association with FEV1: log of odds ratio for FEV1 per effect allele, allele associated with longer telomere length; Per allele association with mean telomere length: SD change in mean telomere length per effect allele. Inverse-variance weighted (IVW), likelihood-based (MaxLik), and MR-Egger (beta) p-values for associations with telomere length and MR-Egger (intercept) for pleiotropy.

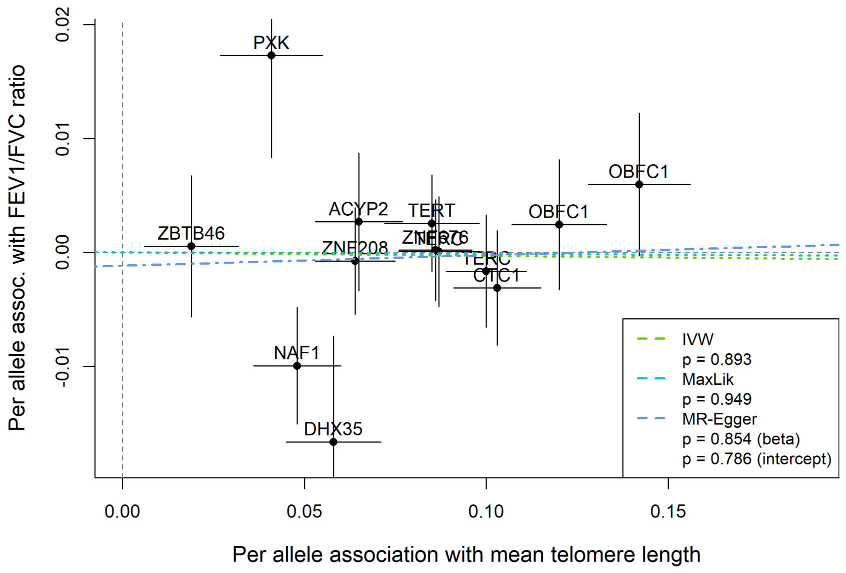

FIG. 13: Per allele association with FEV1/FVC: log of odds ratio for FEV1/FVC per effect allele, allele associated with longer telomere length; Per allele association with mean telomere length: SD change in mean telomere length per effect allele. Inverse-variance weighted (IVW), likelihood-based (MaxLik), and MR-Egger (beta) p-values for associations with telomere length and MR-Egger (intercept) for pleiotropy.

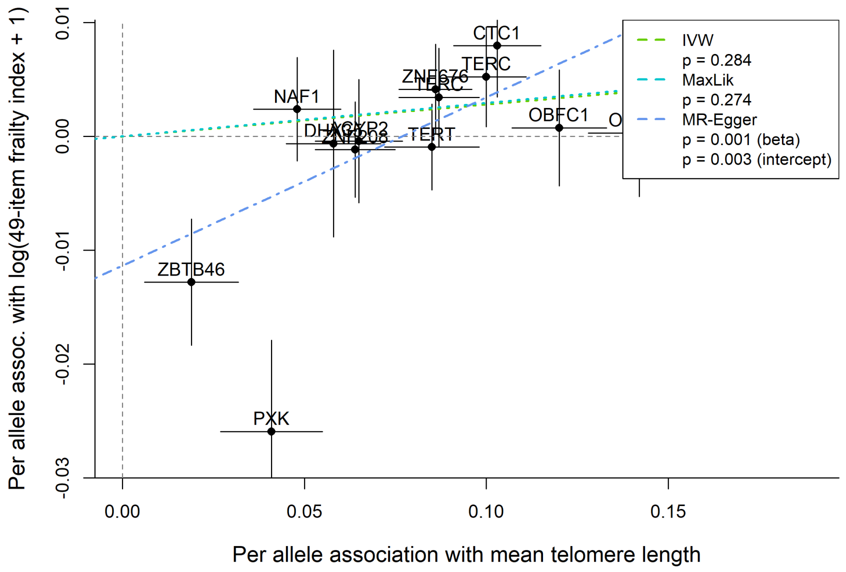

FIG. 14: Per allele association with log(49-item frailty index+1): log of odds ratio for log of 49-item frailty index per effect allele, allele associated with longer telomere length; Per allele association with mean telomere length: SD change in mean telomere length per effect allele. Inverse-variance weighted (IVW), likelihood-based (MaxLik), and MR-Egger (beta) p-values for associations with telomere length and MR-Egger (intercept) for pleiotropy.

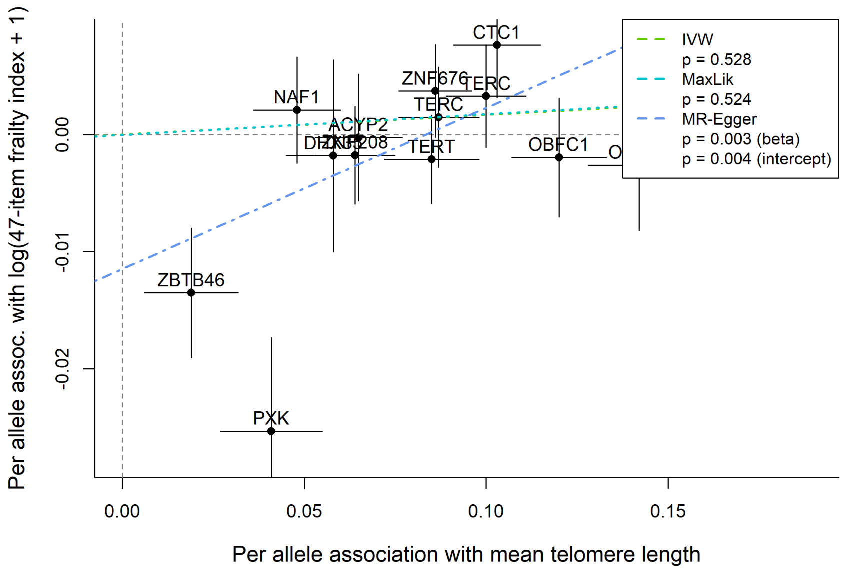

FIG. 15: Per allele association with log(47-item frailty index+1): log of odds ratio for log of 47-item frailty index (excluding two cancer-related items) per effect allele, allele associated with longer telomere length; Per allele association with mean telomere length: SD change in mean telomere length per effect allele. Inverse-variance weighted (IVW), likelihood-based (MaxLik), and MR-Egger (beta) p-values for associations with telomere length and MR-Egger (intercept) for pleiotropy.

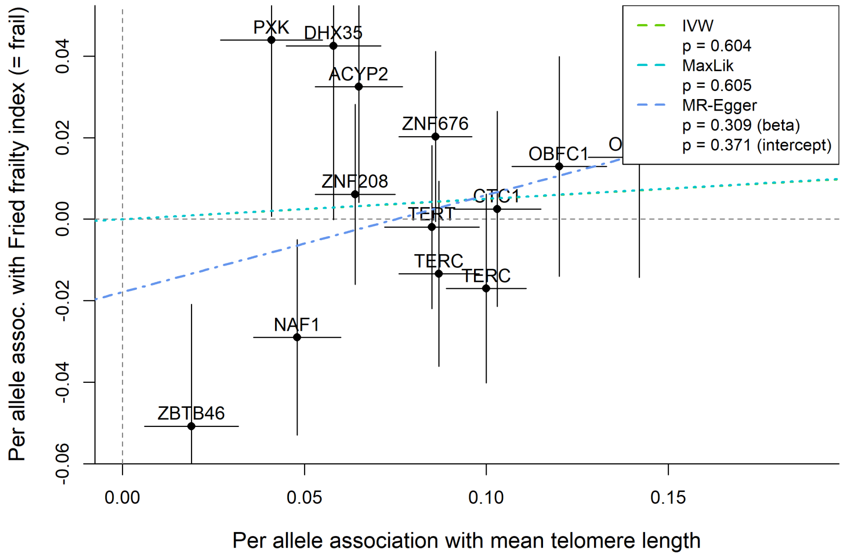

FIG. 16: Per allele association with Fried frailty index: log of odds ratio for Fried frailty index per effect allele, allele associated with longer telomere length; Per allele association with mean telomere length: SD change in mean telomere length per effect allele. Inverse-variance weighted (IVW), likelihood-based (MaxLik), and MR-Egger (beta) p-values for associations with telomere length and MR-Egger (intercept) for pleiotropy.

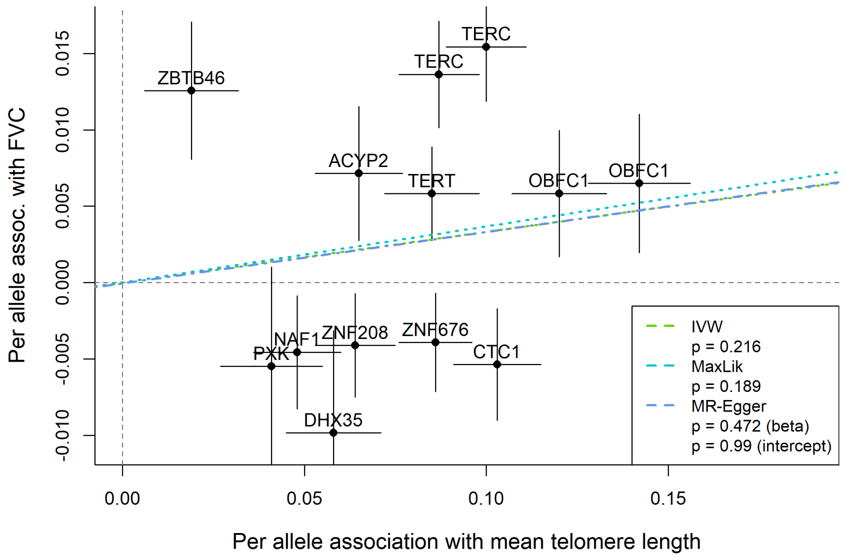

FIG. 17: Per allele association with FVC: log of odds ratio for FVC per effect allele, allele associated with longer telomere length; Per allele association with mean telomere length: SD change in mean telomere length per effect allele. Inverse-variance weighted (IVW), likelihood-based (MaxLik), and MR-Egger (beta) p-values for associations with telomere length and MR-Egger (intercept) for pleiotropy.

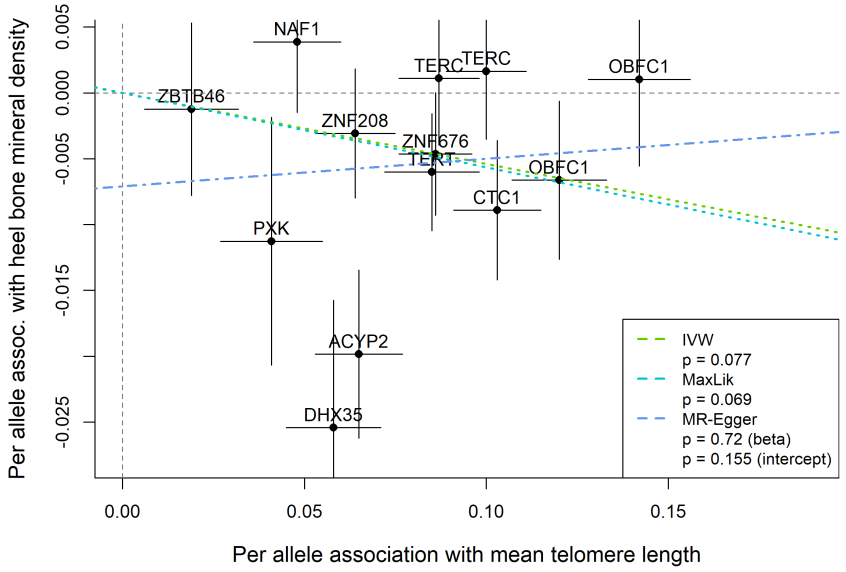

FIG. 18: Per allele association with heel bone mineral density: log of odds ratio for heel bone mineral density per effect allele, allele associated with longer telomere length; Per allele association with mean telomere length: SD change in mean telomere length per effect allele. Inverse-variance weighted (IVW), likelihood-based (MaxLik), and MR-Egger (beta) p-values for associations with telomere length and MR-Egger (intercept) for pleiotropy.

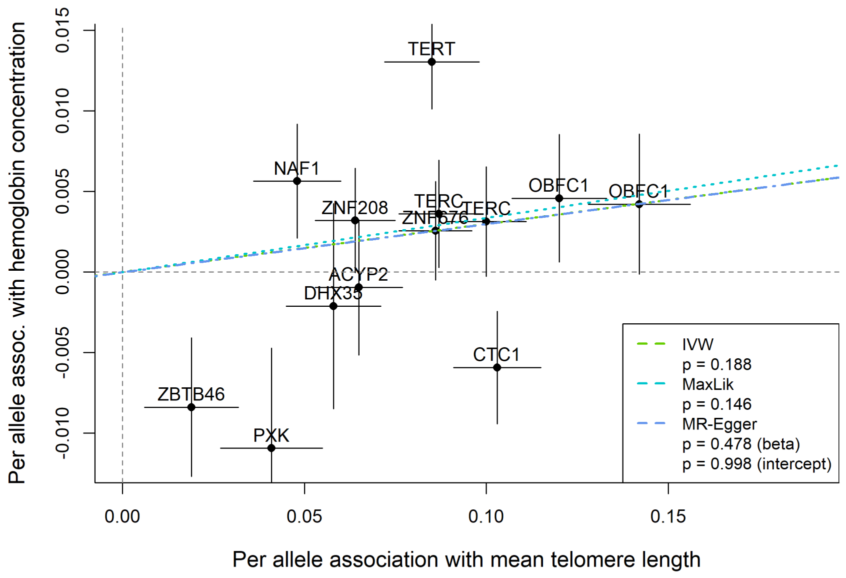

FIG. 19: Per allele association with hemoglobin concentration: log of odds ratio for hemoglobin concentration per effect allele, allele associated with longer telomere length; Per allele association with mean telomere length: SD change in mean telomere length per effect allele. Inverse-variance weighted (IVW), likelihood-based (MaxLik), and MR-Egger (beta) p-values for associations with telomere length and MR-Egger (intercept) for pleiotropy.

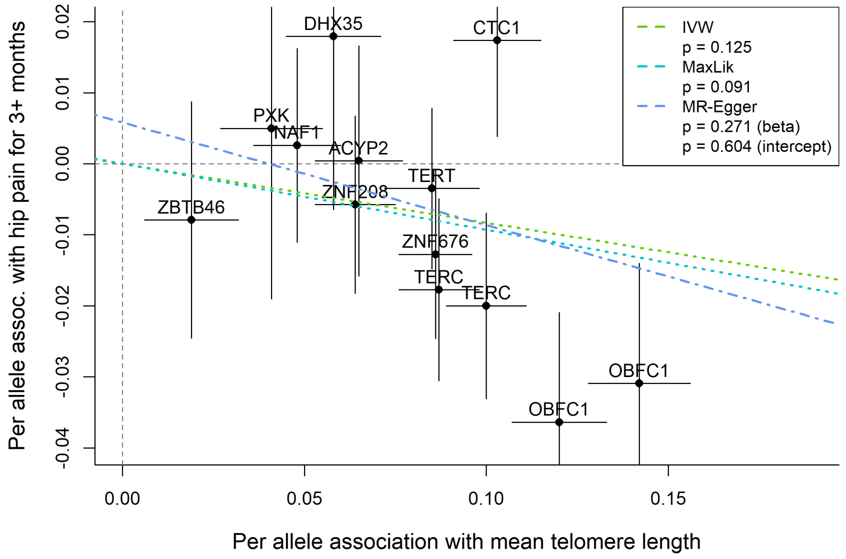

FIG. 20: Per allele association with hip pain for 3+ months: log of odds ratio for hip pain for 3+ months per effect allele, allele associated with longer telomere length; Per allele association with mean telomere length: SD change in mean telomere length per effect allele. Inverse-variance weighted (IVW), likelihood-based (MaxLik), and MR-Egger (beta) p-values for associations with telomere length and MR-Egger (intercept) for pleiotropy.

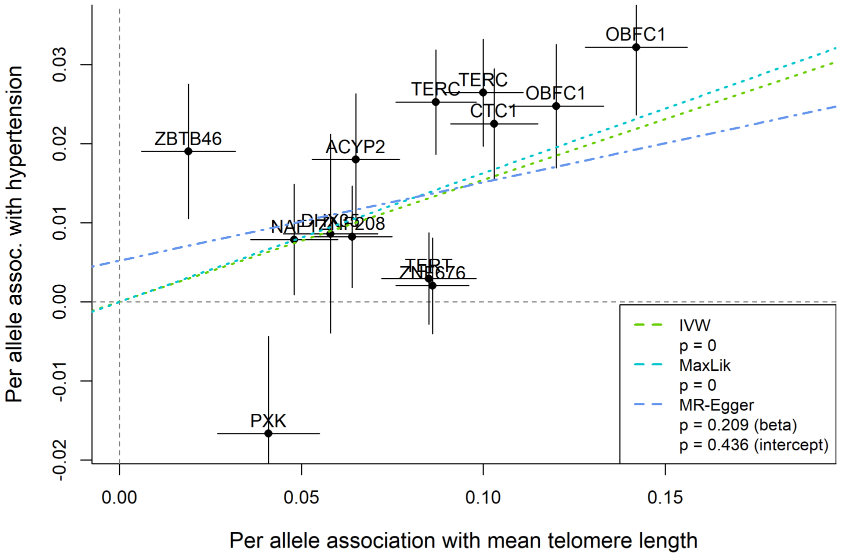

FIG. 21: Per allele association with hypertension: log of odds ratio for hypertension per effect allele, allele associated with longer telomere length; Per allele association with mean telomere length: SD change in mean telomere length per effect allele. Inverse-variance weighted (IVW), likelihood-based (MaxLik), and MR-Egger (beta) p-values for associations with telomere length and MR-Egger (intercept) for pleiotropy.

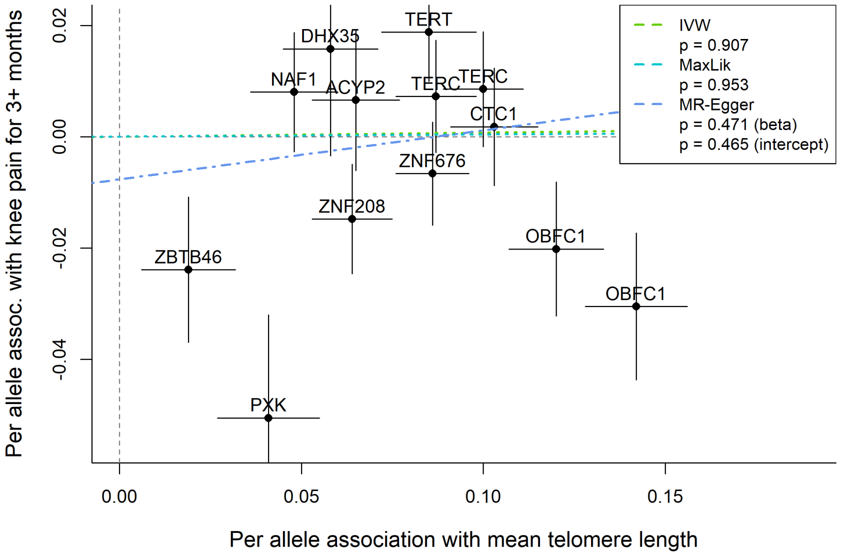

FIG. 22: Per allele association with knee pain for 3+ months: log of odds ratio for knee pain for 3+ months per effect allele, allele associated with longer telomere length; Per allele association with mean telomere length: SD change in mean telomere length per effect allele. Inverse-variance weighted (IVW), likelihood-based (MaxLik), and MR-Egger (beta) p-values for associations with telomere length and MR-Egger (intercept) for pleiotropy.

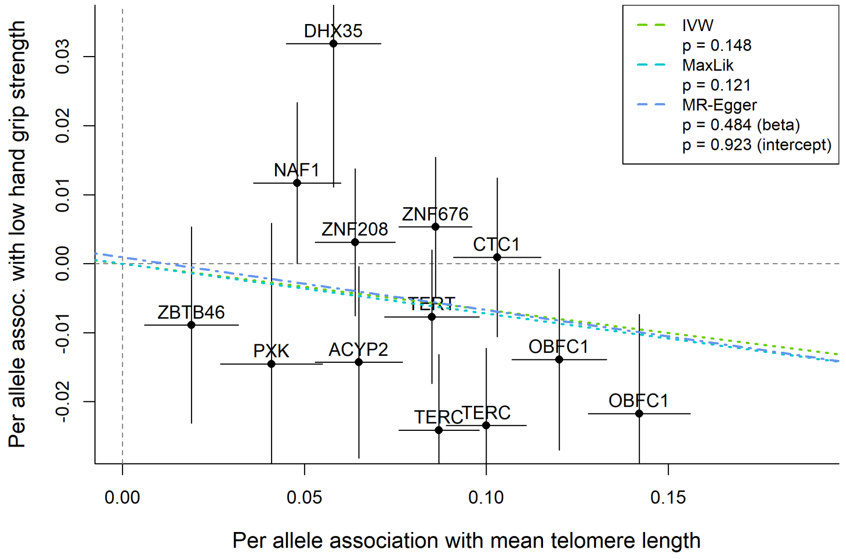

FIG. 23: Per allele association with low hand grip strength: log of odds ratio for low hand grip strength per effect allele, allele associated with longer telomere length; Per allele association with mean telomere length: SD change in mean telomere length per effect allele. Inverse-variance weighted (IVW), likelihood-based (MaxLik), and MR-Egger (beta) p-values for associations with telomere length and MR-Egger (intercept) for pleiotropy.

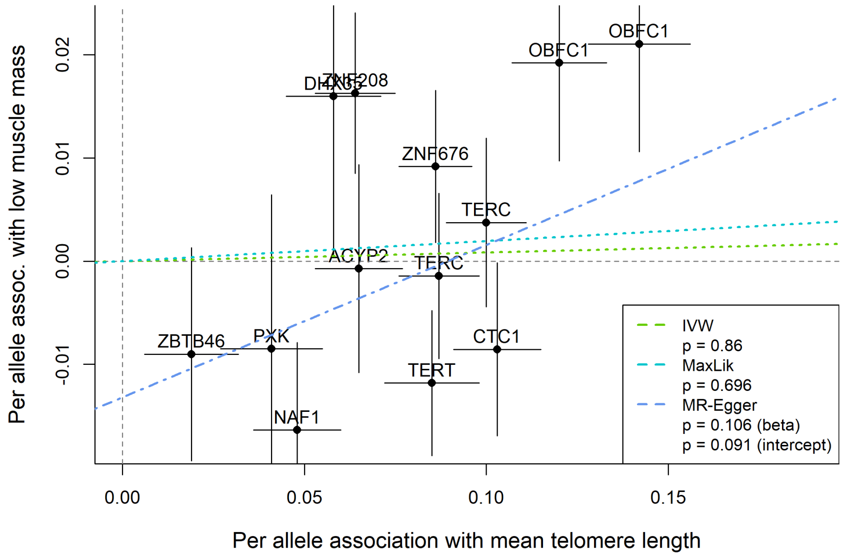

FIG. 24: Per allele association with low muscle mass: log of odds ratio for low muscle mass per effect allele, allele associated with longer telomere length; Per allele association with mean telomere length: SD change in mean telomere length per effect allele. Inverse-variance weighted (IVW), likelihood-based (MaxLik), and MR-Egger (beta) p-values for associations with telomere length and MR-Egger (intercept) for pleiotropy.

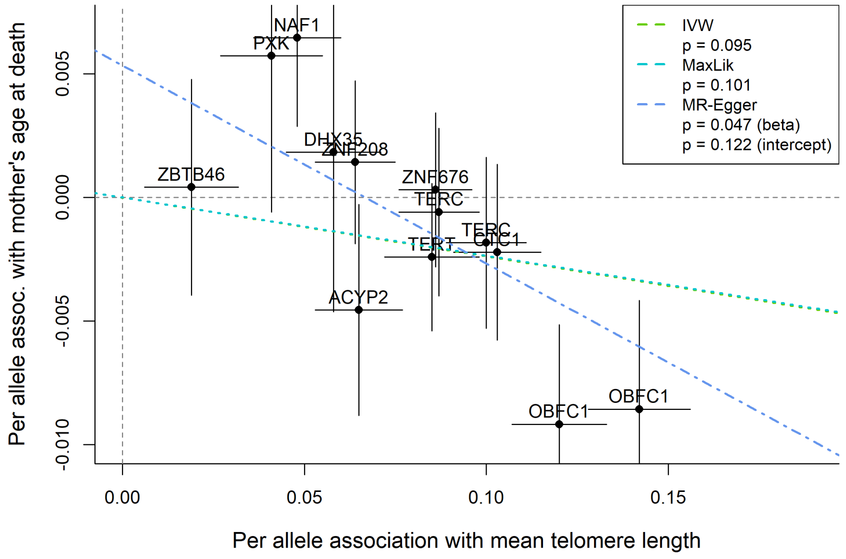

FIG. 25: Per allele association with mother's age at death: log of odds ratio for mother's age at death per effect allele, allele associated with longer telomere length; Per allele association with mean telomere length: SD change in mean telomere length per effect allele. Inverse-variance weighted (IVW), likelihood-based (MaxLik), and MR-Egger (beta) p-values for associations with telomere length and MR-Egger (intercept) for pleiotropy.

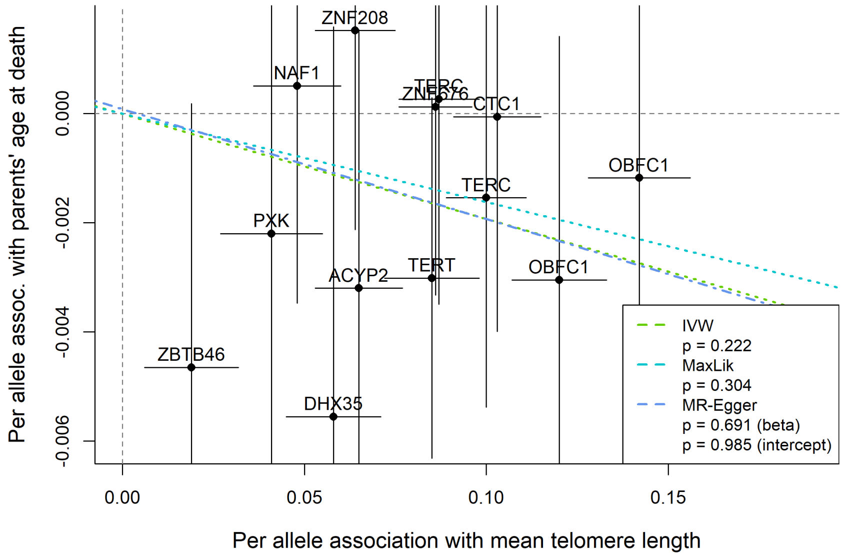

FIG. 26: Per allele association with parents' age at death: log of odds ratio for parents' age at death (average of z-transformed father's and mother's age at death) per effect allele, allele associated with longer telomere length; Per allele association with mean telomere length: SD change in mean telomere length per effect allele. Inverse-variance weighted (IVW), likelihood-based (MaxLik), and MR-Egger (beta) p-values for associations with telomere length and MR-Egger (intercept) for pleiotropy.

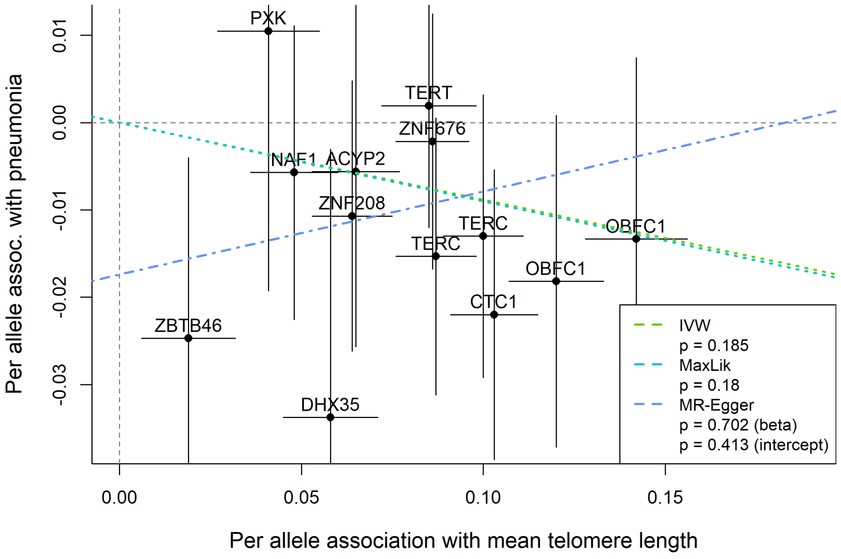

FIG. 27: Per allele association with pneumonia: log of odds ratio for pneumonia per effect allele, allele associated with longer telomere length; Per allele association with mean telomere length: SD change in mean telomere length per effect allele. Inverse-variance weighted (IVW), likelihood-based (MaxLik), and MR-Egger (beta) p-values for associations with telomere length and MR-Egger (intercept) for pleiotropy.

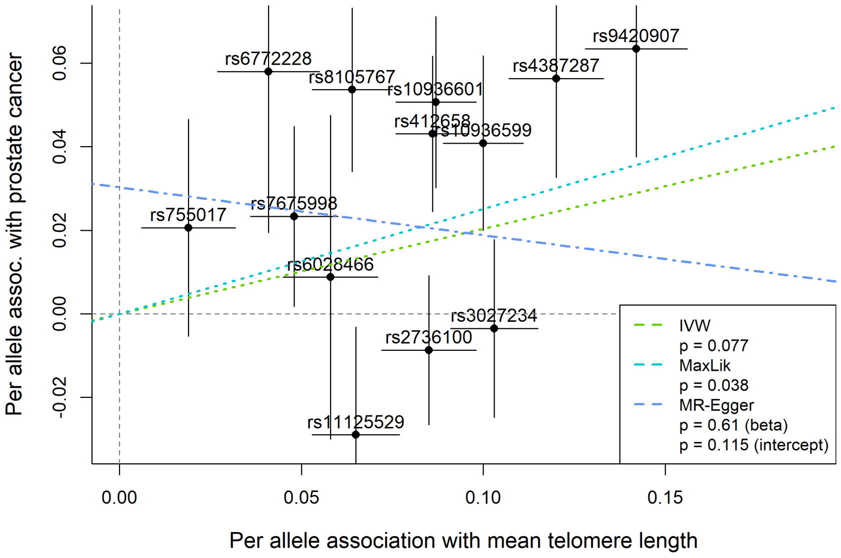

FIG. 28: Per allele association with prostate cancer: log of odds ratio for log of prostate cancer per effect allele, allele associated with longer telomere length; Per allele association with mean telomere length: SD change in mean telomere length per effect allele. Inverse-variance weighted (IVW), likelihood-based (MaxLik), and MR-Egger (beta) p-values for associations with telomere length and MR-Egger (intercept) for pleiotropy.

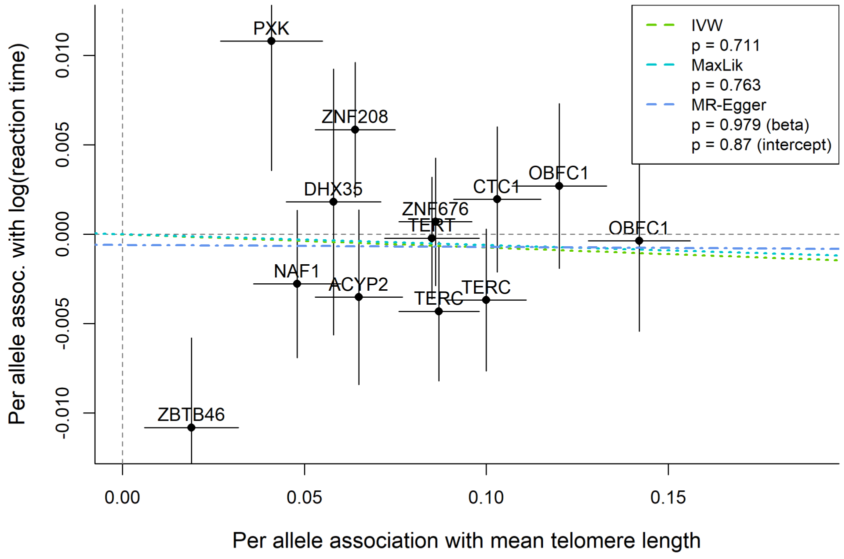

FIG. 29: Per allele association with log(reaction time): log of odds ratio for log of reaction time per effect allele, allele associated with longer telomere length; Per allele association with mean telomere length: SD change in mean telomere length per effect allele. Inverse-variance weighted (IVW), likelihood-based (MaxLik), and MR-Egger (beta) p-values for associations with telomere length and MR-Egger (intercept) for pleiotropy.

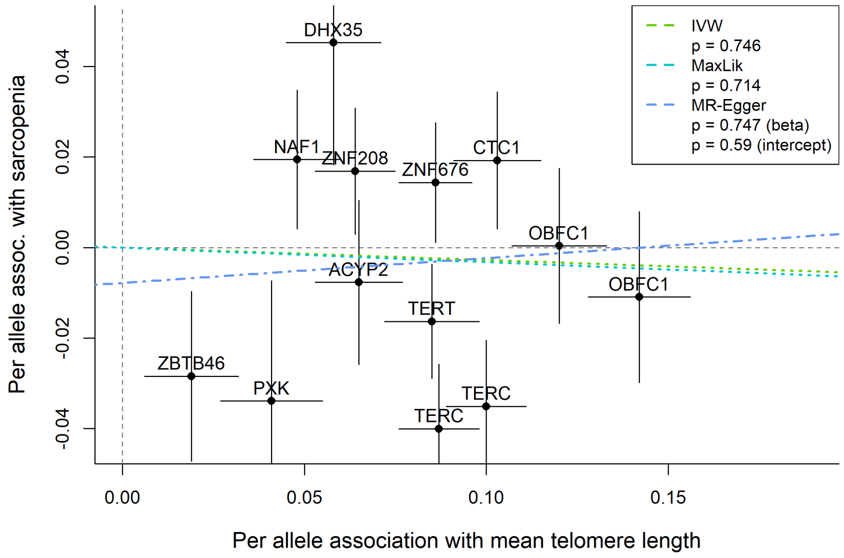

FIG. 30: Per allele association with sarcopenia: log of odds ratio for sarcopenia per effect allele, allele associated with longer telomere length; Per allele association with mean telomere length: SD change in mean telomere length per effect allele. Inverse-variance weighted (IVW), likelihood-based (MaxLik), and MR-Egger (beta) p-values for associations with telomere length and MR-Egger (intercept) for pleiotropy.

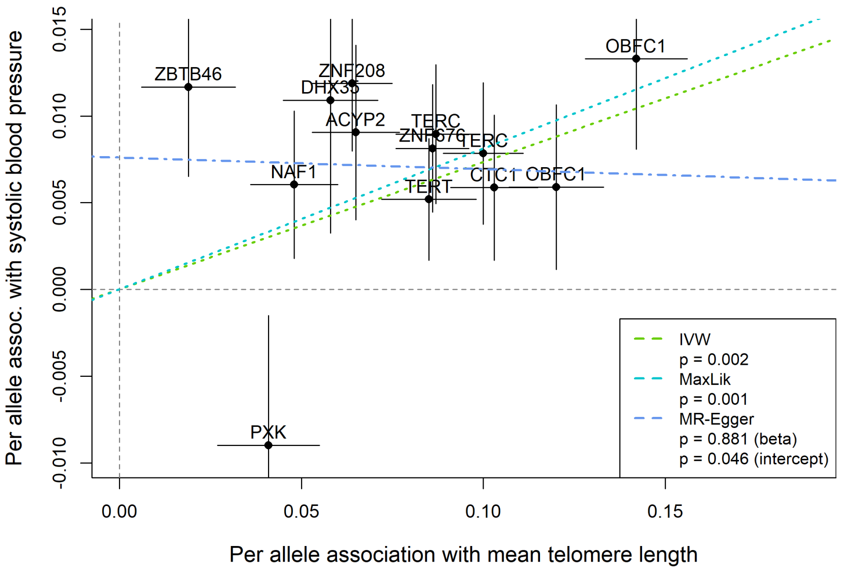

FIG. 31: Per allele association with systolic blood pressure: log of odds ratio for systolic blood pressure per effect allele, allele associated with longer telomere length; Per allele association with mean telomere length: SD change in mean telomere length per effect allele. Inverse-variance weighted (IVW), likelihood-based (MaxLik), and MR-Egger (beta) p-values for associations with telomere length and MR-Egger (intercept) for pleiotropy.

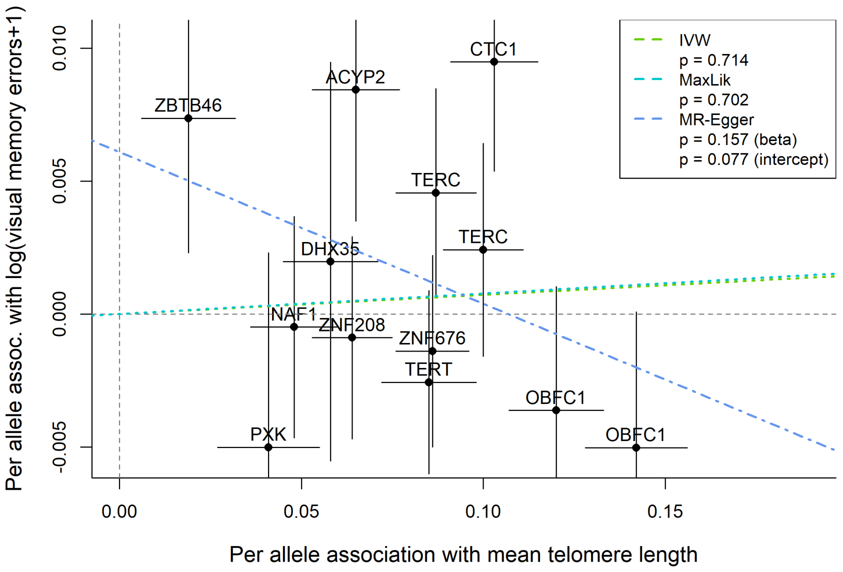

FIG. 32: Per allele association with log(visual memory errors+1): log of odds ratio for visual memory errors per effect allele, allele associated with longer telomere length; Per allele association with mean telomere length: SD change in mean telomere length per effect allele. Inverse-variance weighted (IVW), likelihood-based (MaxLik), and MR-Egger (beta) p-values for associations with telomere length and MR-Egger (intercept) for pleiotropy.
